# Structural Basis for a Safety-Belt Mechanism That Anchors Condensin to Chromosomes

**DOI:** 10.1016/j.cell.2017.09.008

**Published:** 2017-10-19

**Authors:** Marc Kschonsak, Fabian Merkel, Shveta Bisht, Jutta Metz, Vladimir Rybin, Markus Hassler, Christian H. Haering

**Affiliations:** 1 Cell Biology and Biophysics Unit, Structural and Computational Biology Unit, European Molecular Biology Laboratory (EMBL), Meyerhofstraße 1, 69117 Heidelberg, Germany; 2 Protein Expression and Purification Core Facility, European Molecular Biology Laboratory (EMBL), Meyerhofstraße 1, 69117 Heidelberg, Germany

**Keywords:** cell division, chromosome organization, chromosome segregation, condensin, DNA-binding, HEAT repeat, loop extrusion, mitosis, SMC, x-ray crystallography

## Abstract

Condensin protein complexes coordinate the formation of mitotic chromosomes and thereby ensure the successful segregation of replicated genomes. Insights into how condensin complexes bind to chromosomes and alter their topology are essential for understanding the molecular principles behind the large-scale chromatin rearrangements that take place during cell divisions. Here, we identify a direct DNA-binding site in the eukaryotic condensin complex, which is formed by its Ycg1^Cnd3^ HEAT-repeat and Brn1^Cnd2^ kleisin subunits. DNA co-crystal structures reveal a conserved, positively charged groove that accommodates the DNA double helix. A peptide loop of the kleisin subunit encircles the bound DNA and, like a safety belt, prevents its dissociation. Firm closure of the kleisin loop around DNA is essential for the association of condensin complexes with chromosomes and their DNA-stimulated ATPase activity. Our data suggest a sophisticated molecular basis for anchoring condensin complexes to chromosomes that enables the formation of large-sized chromatin loops.

## Highlights

• Condensin Brn1 kleisin and Ycg1 HEAT-repeat subunits create a DNA-binding groove
• DNA is fastened in the groove via its entrapment by a kleisin loop “safety belt”
• Belt closure is required for condensin ATPase activation and chromosome association
• DNA anchoring could provide the basis for condensin-mediated chromatin loop formation

## Graphical Abstract

**Figure:**
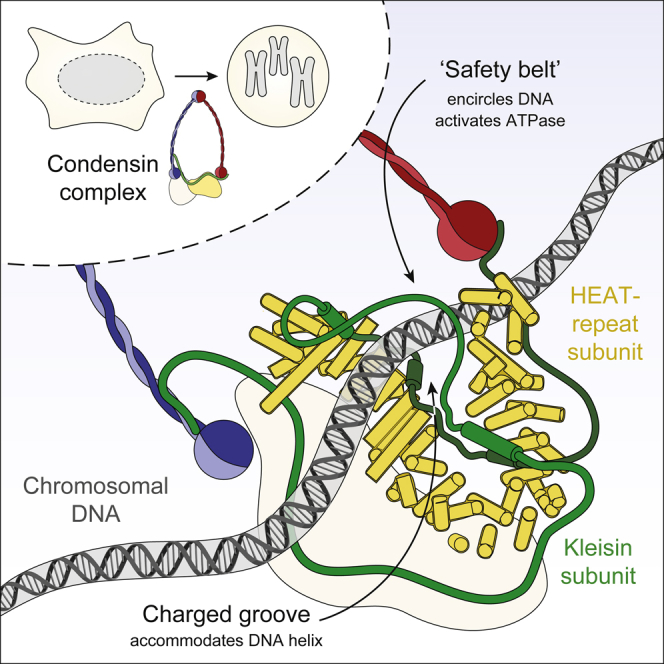

## Teaser

Condensin uses a peptide strap to anchor dsDNA to itself, providing a possible basis for DNA loop formation.

## Introduction

In preparation for cell divisions, eukaryotic chromosomes undergo large-scale conformational changes to form rod-shaped structures that enable their successful segregation into the daughter cells (Hirano et al., 1997, Kschonsak and Haering, 2015). Multisubunit protein complexes named condensins have been recognized as the major molecular machines that coordinate these changes in genome architecture (Houlard et al., 2015, Uhlmann, 2016). Condensins furthermore fulfill pivotal roles in many other aspects of nuclear function, including the regulation of gene expression, during interphase (Hirano, 2016, Rana and Bosco, 2017, Wood et al., 2010).

**Figure 1:**
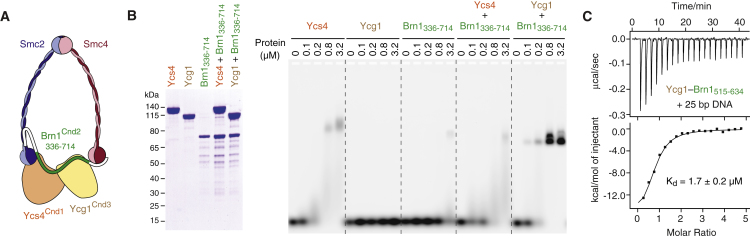
DNA Binding by the Ycg1–Brn1 Condensin Subcomplex (A) Schematic representation of the five-subunit condensin complex. (B) *Ct* His_6_-TEV-Ycs4_3–1222_, His_6_-TEV-Ycg1_24–1006_, and GST*-*HRV3C-Brn1_336–714_ proteins or equimolar combinations of the individually purified proteins were analyzed by SDS-PAGE and Coomassie staining (left panel) or used for EMSA of a 6-fluorescein-amidite (FAM)-labeled 35-bp dsDNA substrate (0.2 μM, right panel). (C) ITC curve of the copurified *Ct* Ycg1_24–1006_–His_6_-TEV-Brn1_515–634_ subcomplex binding to a 25-bp dsDNA. See also Figure S1.

Like other members of the structural maintenance of chromosomes (SMC) family of protein complexes, condensins are characterized by a large, ring-shaped architecture (Anderson et al., 2002, Onn et al., 2007). The condensin ring is formed by heterodimerization of its Smc2 and Smc4 subunits via globular “hinge” domains, which are located at one end of ∼40-nm-long intramolecular antiparallel coiled coils, and the connection of ATP-binding cassette (ABC)-transporter-like ATPase domains at the other end of the coils by the Brn1^Cnd2, NCAPH/H2^ kleisin subunit (Figure 1A). The central region of the kleisin recruits to the condensin ring the Ycs4^Cnd1, NCAPD2/D3^ and Ycg1^Cnd3, NCAPG/G2^ subunits, which contain tandem repeats of amphipathic α helices referred to as HEAT (huntingtin, elongation factor 3, protein phosphatase 2A, Tor1 kinase) motifs (Andrade and Bork, 1995, Neuwald and Hirano, 2000).

Condensin rings, like the related cohesin and prokaryotic SMC complexes, are thought to encircle chromosomal DNA (Cuylen et al., 2011, Ivanov and Nasmyth, 2005, Wilhelm et al., 2015). This topological mode of DNA binding might form the basis for the creation of large chromatin loops, their maintenance, or both (Dekker and Mirny, 2016, Nasmyth, 2001). Recent polymer dynamics simulations demonstrated that loop extrusion by condensin can, at least in principle, recapitulate the formation of cylindrical mitotic chromosomes *in silico* (Goloborodko et al., 2016) and produce structures that are consistent with electron micrographs and chromosome-conformation-capture contact maps of mitotic chromosomes (Earnshaw and Laemmli, 1983, Naumova et al., 2013). Yet, it is difficult to imagine how mere topological entrapment of chromatin fibers within ring-shaped protein complexes could conceivably result in the creation of loops of several kilobase pairs in size or achieve the active compaction of DNA substrates observed in magnetic tweezers experiments (Eeftens et al., 2017, Strick et al., 2004).

It hence seems inevitable that condensin needs to make direct contact with DNA. *In vitro* DNA-binding experiments suggest that the Smc2–Smc4 dimerization hinge interface is able to bind to short, single-stranded, but surprisingly not to double-stranded (ds), DNA molecules (Griese et al., 2010). In contrast, a “non-SMC” subcomplex composed of the central region of the kleisin and HEAT-repeat subunits binds double-stranded, but not single-stranded, DNA (Piazza et al., 2014). The non-SMC subcomplex plays an integral role in the association of condensin with chromosomes, since chromosomal localization of complexes that lack either HEAT-repeat subunit is largely restricted to the axes of chromosomes assembled in *Xenopus laevis* egg extract (Kinoshita et al., 2015) and complexes without the Ycg1 HEAT-repeat subunit fail to associate with mitotic chromosomes in budding yeast and human cells (Piazza et al., 2014). Nevertheless, the mechanistic basis for the loading of condensin complexes onto chromosomes and the role of the HEAT-repeat subunits in this process have remained unknown.

Here, we unveil a direct DNA interaction site in the non-SMC subcomplex, which is formed by the Ycg1 HEAT-repeat and Brn1 kleisin subunits. Co-crystal structures of Ycg1–Brn1 with and without DNA duplexes at near-atomic resolution reveal a conserved, positively charged groove. DNA bound within the groove is locked into place by its entrapment by a peptide loop of the kleisin subunit. We demonstrate the contributions of groove and kleisin loop for condensin binding to DNA *in vitro* and to mitotic chromosomes *in vivo*, reveal its function in the DNA-dependent stimulation of the Smc2–Smc4 ATPase activity, and identify a regulatory mechanism for loop closure in human cells. Our findings suggest a “safety-belt” mechanism that enables condensin to stably bind chromosomes independent of DNA sequence and provides a possibility for condensin to form chromatin loops.

## Results

### Condensin’s Ycg1–Brn1 Subcomplex Binds DNA

**Figure S1:**
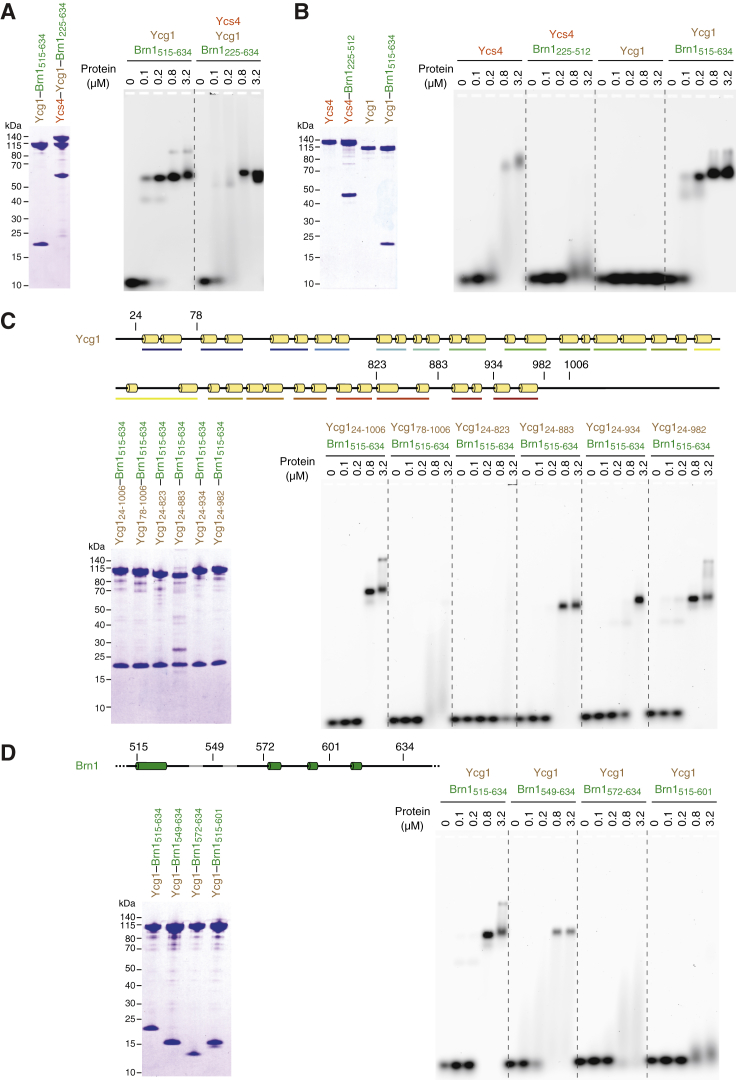
Condensin Subunits and Protein Domains Required for DNA Binding, Related to Figure 1 (A) EMSA with 6-FAM labeled 35-bp dsDNA (0.2 μM) and copurified *Ct* Ycg1_24-1006_- His_6_-TEV-Brn1_515-634_ and *Ct* Ycs4_3-1222_–Ycg1_24-1006_–His_6_-TEV-Brn1_225-634_ subcomplexes. Protein preparations used for EMSA are shown after SDS-PAGE and Coomassie staining. (B) EMSA with *Ct* Ycs4, *Ct* Ycg1 proteins or copurified *Ct* Ycs4–Brn1_225-512_ or *Ct* Ycg1–Brn1_515-634_ subcomplexes as in (A). (C) EMSA with copurified *Ct* Ycg1–His_6_-TEV-Brn1_515-634_ subcomplexes containing truncated versions of *Ct* Ycg1 (Ycg1_24-1006_, Ycg1_78-1006_, Ycg1_24-823_, Ycg1_24-883_, Ycg1_24-934_, Ycg1_24-982_) as in (A). Cartoons indicate truncations of Ycg1 secondary structure elements. (D) EMSA with copurified *Ct* Ycg1_24-1006_– His_6_-TEV-Brn1 subcomplexes containing truncated versions of *Ct* Brn1 (Brn1_515-634_, Brn1_549-634_, Brn1_572-634_, Brn1_515-601_) as in (A). Cartoons indicate truncations of Brn1 secondary structure elements.

Even though the non-SMC subcomplex of condensin binds dsDNA (Piazza et al., 2014), none of its proteins contain conventional DNA-binding motifs. To narrow down the position of the DNA interaction site, we expressed and purified *Chaetomium thermophilum* (*Ct*) Ycs4, Ycg1, and Brn1 subunits individually and tested their binding to a 35-bp DNA substrate in an electrophoretic mobility shift assay (EMSA; Figure 1B). None of the individual subunits reproduced the distinct DNA upshift that we had observed with the *Ct* Ycs4–Ycg1–Brn1 non-SMC subcomplex (Figure S1A). Since Ycs4- and Ycg1-HEAT-repeat subunits do not stably interact with each other directly (Onn et al., 2007), DNA binding might require complex formation between the Brn1 kleisin and either, or possibly both, of the HEAT-repeat subunits. The weak DNA binding by the purified *Ct* Ycs4 protein was, however, reduced—rather than enhanced—by addition of a purified fragment of *Ct* Brn1 that binds to both HEAT-repeat subunits (*Ct* Brn1_336–714_; Figure 1B). A copurified complex of *Ct* Ycs4 and the region of *Ct* Brn1 that binds specifically to this HEAT-repeat subunit (*Ct* Ycs4–Brn1_225–512_) similarly failed to shift DNA efficiently (Figure S1B).

In contrast, addition of *Ct* Brn1_336–714_ to *Ct* Ycg1 resulted in a distinct DNA upshift (Figure 1B). We observed a similarly effective DNA upshift with a copurified subcomplex of Ycg1 and its interacting region of Brn1 (*Ct* Ycg1–Brn1_515–634_; Figure S1B), which equaled the upshift measured for the *Ct* Ycs4–Ycg1–Brn1 non-SMC complex (Figure S1A). Quantitation of the DNA interaction of the *Ct* Ycg1–Brn1_515–634_ subcomplex by isothermal titration calorimetry (ITC; Figure 1C) revealed a dissociation constant *K*_*d*_ of 1.7 ± 0.2 μM, which is identical to the affinity we had previously measured for the Ycs4–Ycg1–Brn1 ternary complex (*K*_*d*_ = 1.7 ± 0.2 μM, Piazza et al., 2014). DNA binding required the presence of the first 16 of 19 HEAT-repeat motifs of Ycg1 (*Ct* Ycg1_24–883_; Figure S1C) and the C-terminal two-thirds of the interacting Brn1 region (*Ct* Brn1_549–634_; Figure S1D). We conclude that the main DNA-binding activity of the non-SMC subcomplex resides within the Ycg1-HEAT-repeat subunit and the region of the Brn1 kleisin subunit it interacts with.

### Structure of the Ycg1–Brn1 Subcomplex

**Figure 2:**
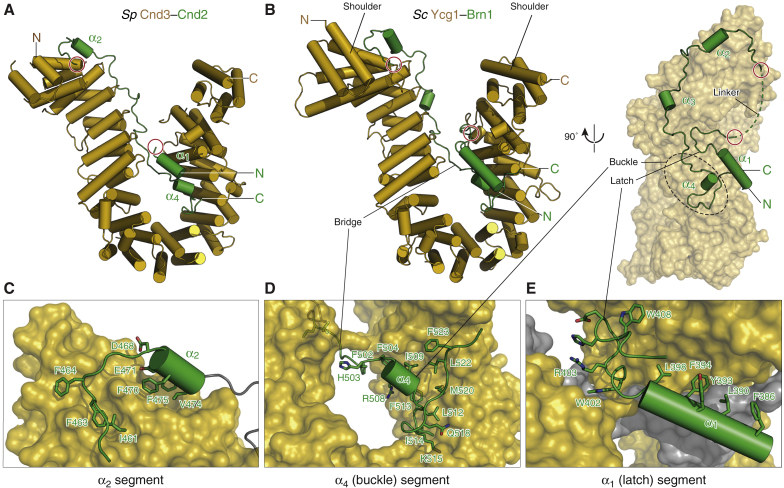
Structures of Ycg1–Brn1 Condensin Subcomplexes (A) Cartoon model of *Sp* Cnd3–Cnd2 subcomplex based on the purified *Sp* Cnd3_1–438, 474–823_–Cnd2_416–544_ construct. N and C termini of the HEAT-repeat subunit (yellow) and kleisin subunit (green) and the ends of a disordered region in the Cnd2 kleisin subunit (red circles) are indicated. (B) Cartoon and surface models of the *Sc* Ycg1–Brn1 subcomplex based on the purified Ycg1_6–498, 556–932_–Brn1_384–529_ construct. Color scheme as in (A). The Brn1 kleisin disordered linker, α_4_ buckle, and α_1_ latch regions are indicated. (C–E) Close-up views of the Brn1 α_2_ (C), α_4_ buckle (D), and α_1_ latch (E) segments in the *Sc* Ycg1–Brn1 subcomplex. See also Figures S2, S3, and S4 and Table S1.

**Figure S2:**
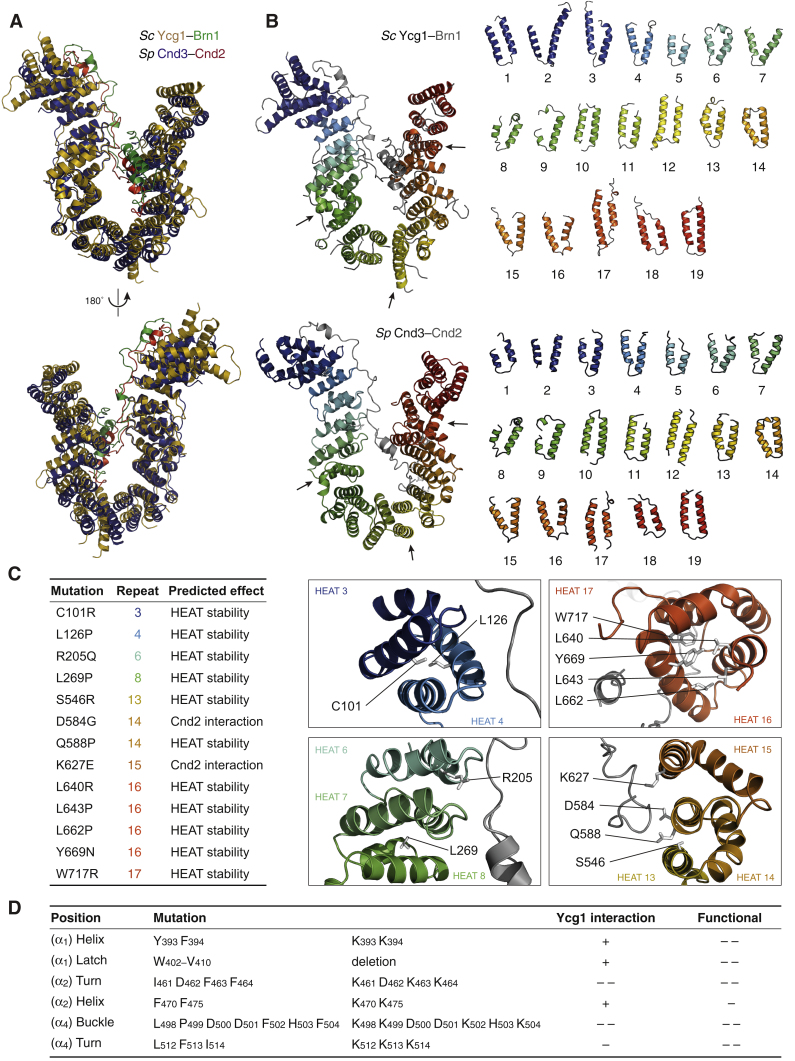
Comparison of *Sp* Cnd3–Cnd2 and *Sc* Ycg1–Brn1 Crystal Structures, Related to Figure 2 (A) Structural alignment of *Sp* Cnd3–Cnd2 (blue, red) and *Sc* Ycg1–Brn1 (yellow, green) over all C_α_ atoms (RMSD 2.53 Å over 715 C_α_). (B) Cartoon representation of the 19 HEAT repeats of *Sc* Ycg1–Brn1 (top) or of *Sp* Cnd3–Cnd2 (bottom). Arrows indicate positions of irregularities between canonical HEAT-repeat stretches. (C) Positions of point mutations in Cnd3 that cause temperature or DNA damage sensitivity in *Sp* (Petrova et al., 2013, Xu et al., 2015) and their predicted effect based on the Cnd3–Cnd2 structure. (D) Summary of the effects of previously identified *Sc* Brn1 mutations on the co-immunoprecipitation of Ycg1 from yeast cell extracts (+ no effect, – reduced Ycg1 co-immunoprecipitation, – – strongly reduced Ycg1 copurification) and on the ability of Brn1 mutant proteins to complement the deletion of the endogenous *BRN1* gene (– reduced growth, – – no growth) (Piazza et al., 2014).

**Figure S3:**
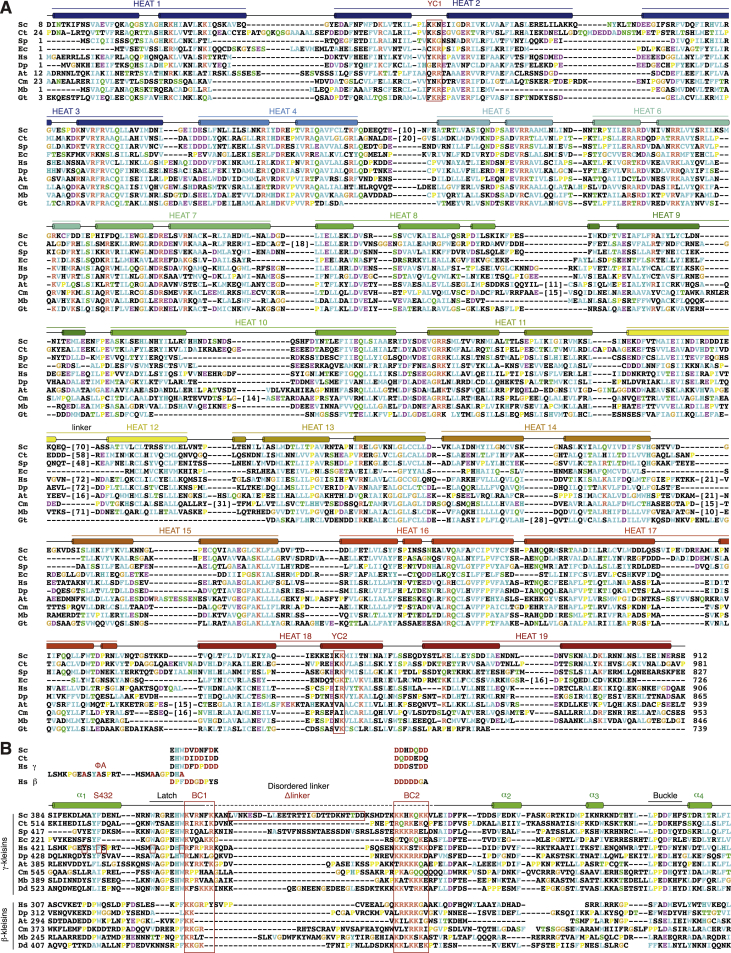
Multi-sequence Alignment of Selected Condensin HEAT-Repeat and Kleisin Sequences, Related to Figures 2 and 3 (A) Sequence alignment of four yeast (*Sc Saccharomyces cerevisiae, Ct Chaetomium thermophilum, Sp Schizosaccharomyces pombe, Ec Encephalitozoon cuniculi*), two animal (*Hs Homo sapiens, Dp Daphnia pulex*), two plant (*At Arabidopsis thaliana, Cm Cyanidioschyzon merolae*) and two protist (*Mb Monosiga brevicollis, Gt Guillardia theta*) species selected from an alignment of sequences from 35 divergent species. Secondary structure elements are highlighted based on the *Sc* Ycg1–Brn1 structure. Sites of mutations in *Sc* Ycg1 that abolish DNA binding (YC1 and YC2) are highlighted by red boxes. (B) Sequence alignments of α- (cohesin) and γ- (condensin) kleisins of four yeast and of α-, β- (condensin II), and γ- (condensin I) kleisins of two animal, two plant, and two protist (*Dd Dictyostelium discoideum*) species selected from an alignment of sequences from 35 divergent species as in (A). Note that yeast genomes encode no β-kleisin subunit. Secondary structure elements are highlighted based on the *Sc* Ycg1–Brn1 structure. DNA binding site mutations (BC1 and BC2), mutations of hydrophobic latch residues (ΦA), the phosphorylated serine residue in NCAPH (S432), and the region that was deleted in the *Sc* Brn1_Δlinker (short loop)_ construct are highlighted by red boxes.

To gain detailed insight into the nature of the DNA binding site, we solved crystal structures of the Ycg1–Brn1 subcomplex of *Saccharomyces cerevisiae* (*Sc*) and of the homologous Cnd3–Cnd2 subcomplex of *Schizosaccharomyces pombe* (*Sp*) to 2.8 Å and 2.6 Å resolution, respectively (Table S1). Both structures are strikingly similar and reveal a harp-shaped conformation of the HEAT-repeat subunit, with the kleisin subunit winding along the concave surface of the HEAT-repeat solenoid (Figures 2A, 2B, and S2A). The peculiar curvature of Ycg1 and Cnd3 has its cause in three discontinuities in the directionality of the solenoid formed by the 19 HEAT-repeat motifs (Figures S2B and S3A). Several residues that have previously been found to destabilize Cnd3 (Petrova et al., 2013, Xu et al., 2015) are involved in contacts between the repeat helices (Figure S2C), which emphasizes the importance of the HEAT-repeat solenoid conformation.

**Figure S4:**
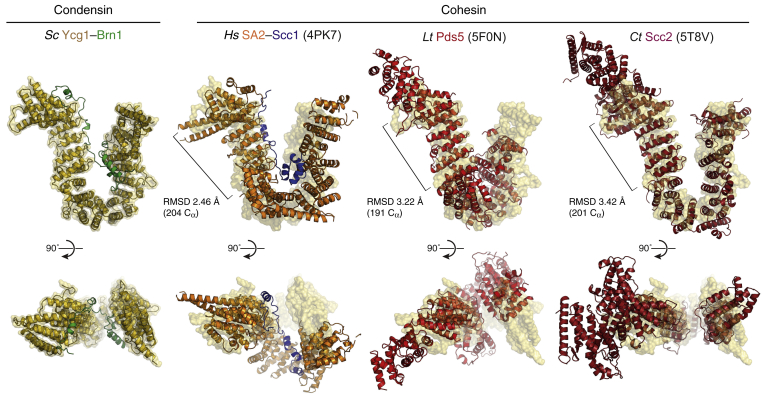
Comparison of the *Sc* Ycg1–Brn1 Structure with Structures of Cohesin HEAT-Repeat Subunits, Related to Figures 2 and 3 Overview and structural alignments of *Sc* Ycg1–Brn1 with crystal structures of cohesin HEAT-repeat subunits *Hs* SA2–Scc1 (PDB: 4PK7), *Ct* Scc2 (PDB: 5T8V) and *Lachancea thermotolerans* (*Lt*) Pds5 (PDB: 5F0N). Alignments were generated by secondary structure matching using only C atoms from *Sc* Ycg1–Brn1 HEAT repeats 2 to 7 and structurally equivalent regions of the cohesin HEAT-repeat subunits.

A comparison with known crystal structures of the cohesin-associated HEAT-repeat subunits SA2, Pds5, and Scc2 (Hara et al., 2014, Kikuchi et al., 2016, Lee et al., 2016) shows that all four proteins have similarly curved shapes. Yet, the relative positions and orientations of the individual HEAT motifs vary notably between Ycg1 and the cohesin HEAT-repeat subunits (Figure S4). It is hence conceivable that these subunits have evolved from a common ancestral protein (Wells et al., 2017) to fulfill distinct functions in cohesin and condensin complexes (see Discussion).

The Brn1 kleisin subunit makes extended contacts with the inner surface of the Ycg1 harp, burying a total surface area of 3,754 Å^2^. Several conserved aromatic residues within a Brn1 segment that contains a short α-helical stretch (α_2_) are involved in hydrophobic interactions with HEAT motifs 1 and 2 of the N-terminal “shoulder” of the harp (Figures 2B and 2C). The next ∼20 residues form a well-ordered linker that contains a short helix (α_3_) and follows the inward-facing ridge of HEAT motifs 3–6 of the N-terminal lobe of Ycg1 before crossing over to the C-terminal lobe of Ycg1. Through multiple hydrophobic, electrostatic, and hydrogen bond interactions with the HEAT-repeat subunit, Brn1 forms a rigid “bridge” between the two Ycg1 lobes (Figures 2B and 2D). The consecutive segment that follows contains another short helix (α_4_) and a conserved coiled region that packs through hydrophobic interactions and hydrogen bonds onto Ycg1 HEAT motifs 13–15 (Figure 2D).

One of the two molecules in the asymmetric unit of the Ycg1–Brn1 and Cnd3–Cnd2 crystals shows additional electron density for a segment that corresponds to a long helix (α_1_) in the N-terminal region of the kleisin subunit (Figures 2A and 2B). This helix and the succeeding well-ordered turn stack onto HEAT motifs 16 and 17 and onto the α_4_ segment of the kleisin via several highly conserved aromatic residues in a manner that resembles a “latch” that has been inserted into a “buckle” (Figures 2B and 2E). Through the interaction of latch and buckle regions, the kleisin subunit adopts a loop configuration (Figure 2B). As we demonstrate below, this loop has important implications for DNA binding.

The absence of density for the latch segment in half of the complexes suggests that the α_2_ to α_4_ segments of the kleisin subunit are sufficient for complex formation with the HEAT-repeat subunit. This notion is consistent with the finding that mutation of patches of conserved hydrophobic residues within the Brn1 α_2_ (*Sc* Brn1_461–464_) or α_4_ (*Sc* Brn1_498–504_ or *Sc* Brn1_512–514_) segments, which make major contacts with the HEAT repeats, disrupt binding to Ycg1 (Figure S2D; Piazza et al., 2014). Mutation or deletion of conserved patches that contribute to the stacking of the N-terminal α_1_ segment of Brn1 (*Sc* Brn1_393–394_ or *Sc* Brn1_402–410_) has, in contrast, little effect on the overall affinity to Ycg1, while nevertheless rendering condensin non-functional. Whereas the latch segment of the kleisin subunit might not be essential for complex formation with the HEAT-repeat subunits, it is essential for condensin function.

### A Composite Ycg1–Brn1 DNA-Binding Groove Is Required for Condensin Localization to Chromosomes

**Figure 3:**
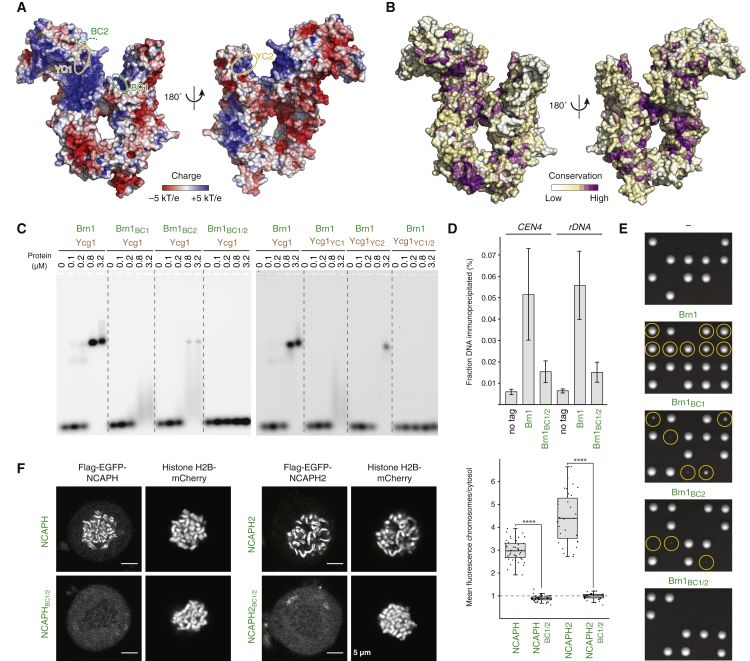
A Conserved Positively Charged Ycg1–Brn1 Groove Is Essential for Condensin’s Association with Chromosomes (A) Electrostatic surface potential representation of the *Sc* Ycg1–Brn1 subcomplex. Regions of positively charged Brn1 (BC1, BC2) or Ycg1 (YC1, YC2) residue patches are indicated. (B) Conservation surface representation of the *Sc* Ycg1–Brn1 subcomplex. Conservation scores were calculated based on an alignment of sequences from 35 evolutionary distant eukaryotic species. (C) EMSA of a 6-FAM-labeled 35-bp dsDNA substrate (0.2 μM) with copurified *Ct* Ycg1_24–1006_–His_6_-TEV-Brn1_515–634_ subcomplexes containing wild-type or charged-patch mutant versions of *Ct* Brn1 (BC1: *Ct* Brn1_R539D, R541D, K542D, K544D_, BC2: *Bc* Brn1_R554D, R556D, K557D, K559D_, BC1/2: *Bc* Brn1_R539D, R541D, K542D, K544D, R554D, R556D, K557D, K559D_) or *Ct* Ycg1 (YC1: *Ct* Ycg1_K100D, K101D_, YC2: *Ct* Ycg1_K916D, K917D_, YC1/2: *Ct* Ycg1_K100D, K101D, K916D, K917D_). (D) ChIP-qPCR of condensin complexes containing wild-type *Sc* Brn1-PK_6_ (strain C4239) or mutant *Sc* Brn1_BC1/2_-PK_6_ (*Sc* Brn1_K409D, R411D, K414D, K451D, K452D, K454D, K456D, K457D_) in asynchronous cells at centromeric (*CEN4*) and *rDNA* genomic loci. Error bars indicate mean ± SD of two independent experiments with two qPCR repeats each. (E) Tetrad dissection of *BRN1*/*brn1Δ* diploid budding yeast cells expressing no (–, strain C4237), wild-type (Brn1, strain C4239), or mutant (BC1: *Sc* Brn1_K409D, R411D, K414D_, strain C4257, BC2: *Sc* Brn1_K451D, K452D, K454D, K456D, K457D_, strain C4259, BC1/2: *Sc* Brn1_K409D, R411D, K414D, K451D, K452D, K454D, K456D, K457D_, strain C4261) versions of Brn1-PK_6_ from an ectopic locus under control of the endogenous promoter. Images were recorded after three days at 25°C. Genetic marker analysis identifies *BRN1*_*x*_, *brn1Δ* cells (circles). (F) Representative example images of nocodazole-arrested HeLa cells expressing mCherry-tagged histone H2B and transiently transfected Flag-EGFP-tagged NCAPH or NCAPH2 as wild-type or charged-patch mutant (BC1/2: *Hs* NCAPH_R446D, R448D, R450D, R451D, K452D, K462D, K463D, K464D, K467D, K468D_, *Hs* NCAPH2_K329D, K332D, K333D, R335D, K350D, R351D, K352D, R353D, K354D_) versions. Scale bars: 5 μm. The graph plots ratios of chromosomal to cytosolic EGFP intensities. Horizontal lines indicate median, hinges indicate first and third quartiles, and whiskers extend to the highest or lowest point from the hinges within 1.5 times interquartile range, calculated from two independent experiments with a total of n = 45 (NCAPH), n = 45 (NCAPH_BC1/2_), n = 31 (NCAPH2), and n = 35 (NCAPH2_BC1/2_) cells (p < 0.0001 by Student’s t test with Welch’s correction). See also Figures S3, S4, and S5 and Table S2.

**Figure S5:**
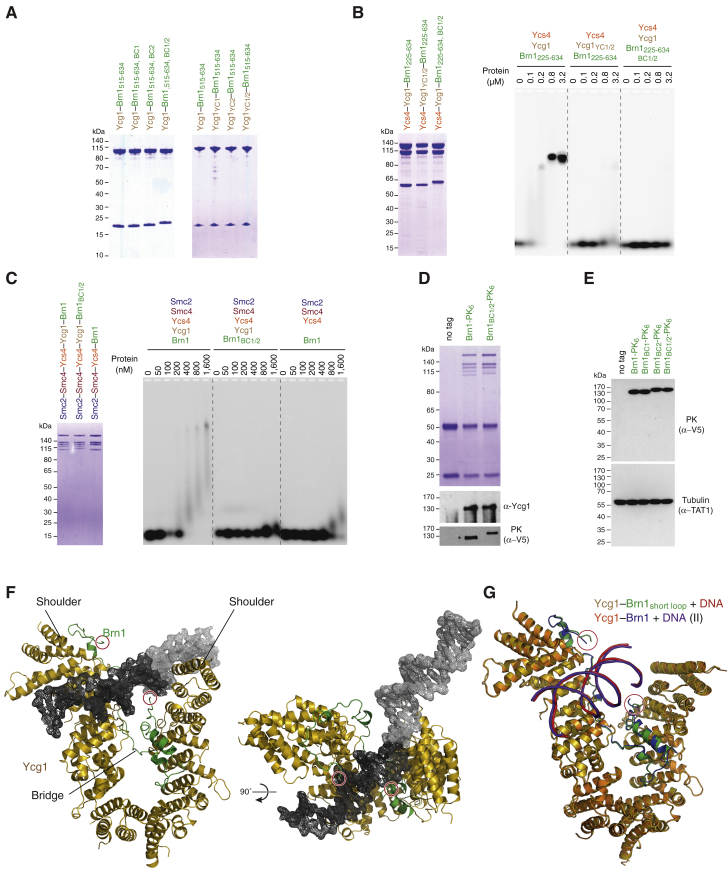
The Ycg1–Brn1 Subcomplex Represents the High-Affinity DNA Binding Site in Condensin, Related to Figures 3 and 4 (A) Coomassie-stained gels of protein complexes used for EMSA (see Figure 3C). (B) EMSA with 6-FAM-labeled 35-bp dsDNA (0.2 μM) and copurified *Ct* Ycs4_3-1222_–Ycg1_24-1006_– His_6_-TEV-Brn1_225-634_ complexes of wild-type, Brn1 mutant BC1/2 (Brn1_R539D, R541D, K542D, K544D, R554D, R556D, K557D, K559D_), or Ycg1 mutant YC1/2 (Ycg1_K100D, K101D, K916D, K917D_). Protein preparations used for EMSA are shown after SDS-PAGE and Coomassie staining. (C) EMSA with 6-FAM-labeled 35-bp dsDNA (0.2 μM) and wild-type or Brn1 BC1/2 (Brn1_K409D, R411D, K414D, K451D, K452D, K454D, K456D, K457D_) mutant *Sc* condensin holocomplexes (Smc2–Smc4-StrepII_3_–Ycs4–Ycg1–Brn1-His_12_-HA_3_) or a tetrameric complex that lacks Ycg1 (Smc2–Smc4-StrepII_3_–Ycs4–Brn1-His_12_-HA_3_). Protein preparations used for EMSA are shown after SDS-PAGE and Coomassie staining. (D) Immunoprecipitation of endogenous condensin complexes of strains C4237, C4239 and C4261 used for ChIP-qPCR (see Figure 3D) analyzed by SDS-PAGE and Coomassie staining or western blotting against Ycg1 or the PK_6_ tag on Brn1. (E) Brn1 expression levels of yeast strains C4237, C4239, C4257, C4259 and C4261 (see Figure 3E) analyzed by western blotting of whole cell lysates against the PK_6_ tag on Brn1 and α-tubulin as loading control. (F) Overview of the *Sc* Ycg1–Brn1 crystal structure in complex with an 18-bp dsDNA (crystal form I). Experimental maps for DNA (dark gray, 1.0 σ) and a symmetry-related 18-bp dsDNA molecule (light gray) are shown. (G) Structural alignment of *Sc* Ycg1–Brn1–DNA (crystal form II) and *Sc* Ycg1–Brn1_short loop_–DNA using all C_α_ atoms (RMSD 0.39 Å over 801 Cα). Free ends of the disordered Brn1 linker are indicated (red circles).

Mapping of the electrostatic surface potential onto the *Sc* Ycg1–Brn1 structure revealed that the wide groove between the two shoulders of the Ycg1 harp and the Brn1 bridge displays a high degree of positive surface charge (Figure 3A). To test whether this groove is involved in DNA binding, we designed charge-reversal mutations for patches of highly conserved basic groove residues located within the N- or C-terminal lobes of Ycg1 (YC1 or YC2) or at either end of the disordered linker of Brn1 (BC1 or BC2; Figures 3A, 3B, S3A, and S3B). Mutation of any of these four patches strongly reduced the affinity of *Ct* Ycg1–Brn1 subcomplexes for DNA *in vitro* (Figures 3C) without affecting protein complex formation (Figure S5A). Combination of the patch mutations in Ycg1 (YC1/2) or Brn1 (BC1/2) even completely abolished any measurable DNA interaction of *Ct* Ycg1–Brn1 or *Ct* Ycs4–Ycg1–Brn1 subcomplexes (Figures 3C and S5B). These findings confirm that the direct protein-DNA binding site in condensin’s non-SMC subcomplex is formed by Ycg1–Brn1, with little or no contribution from the other HEAT-repeat subunit. Mutation of Brn1 BC1/2 residues was even sufficient to abolish DNA binding by condensin holocomplexes, which, like complexes lacking the Ycg1 subunit, failed to shift DNA *in vitro* (Figure S5C). We conclude that the Ycg1–Brn1 groove forms an essential protein-DNA interaction site in condensin complexes.

If this conclusion were correct, condensin complexes with mutations in the DNA binding groove should no longer be able to associate with chromosomes *in vivo*. Indeed, mutation of both positively charged patches in Brn1 (BC1/2) substantially reduced the levels of condensin on chromosomes when measured by chromatin immunoprecipitation followed by quantitative PCR (ChIP-qPCR) at two independent binding sites in the budding yeast genome (Figure 3D), although the mutations did not affect the assembly of condensin holocomplexes (Figure S5D). As expected, cells expressing the BC1/2 double mutant version as their only source of Brn1 failed to divide, and cells expressing only BC1 or BC2 single mutant versions displayed proliferation defects (Figure 3E) despite producing the mutant Brn1 proteins at wild-type levels (Figure S5E).

Finally, we tested whether DNA binding by the HEAT-repeat and kleisin subunits is a conserved feature in the two distinct condensin complexes found in most metazoan organisms. We introduced BC1/2 mutations into the NCAPH or NCAPH2 kleisin subunits of *Homo sapiens* (*Hs*) condensin I or II, respectively (Figure S3B). Whereas wild-type versions of NCAPH or NCAPH2 fused to enhanced green fluorescent protein (EGFP) distinctively stained the axes of mitotic chromosomes in live cells, mutant versions of either kleisin subunit remained exclusively cytoplasmic (Figure 3F). We conclude that DNA binding by the Ycg1–Brn1 groove is essential for the stable association of condensin complexes with chromosomes and consequently condensin function.

### Structure of the Ycg1–Brn1 Subcomplex Bound to DNA

**Figure 4:**
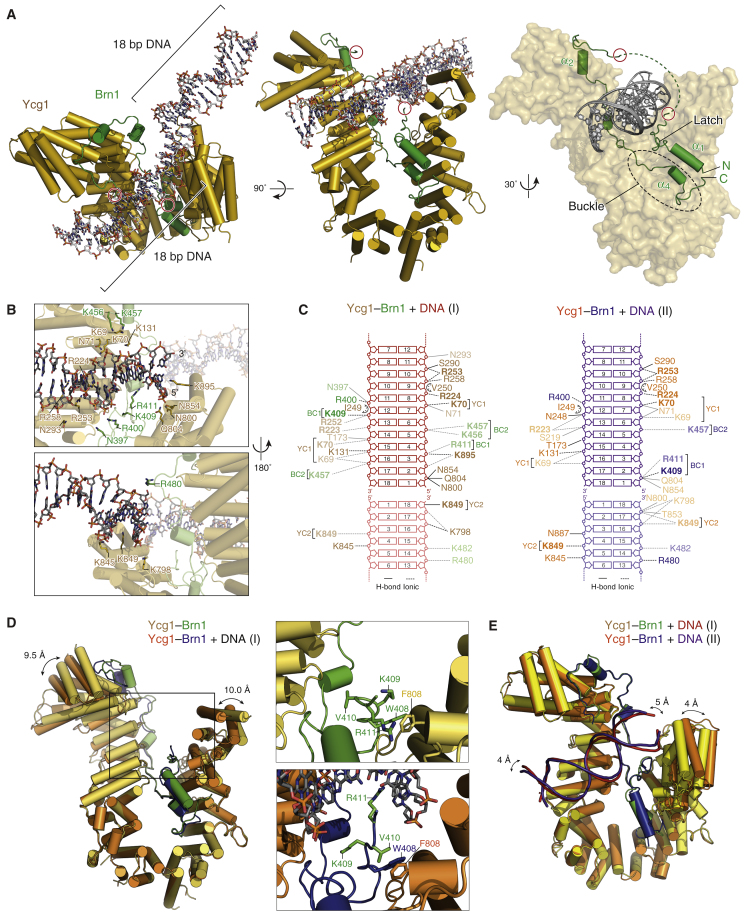
Crystal Structure of Ycg1–Brn1 in Complex with DNA (A) Cartoon and surface models of the *Sc* Ycg1–Brn1 complex bound to an 18-bp dsDNA substrate and to the 18-bp dsDNA of its symmetry-related neighbor, based on the *Sc* Ycg1_6–498, 556–932_–Brn1_384–529_ construct. The ends of the disordered region of *Sc* Brn1 (red circles), the Brn1 kleisin disordered linker, α_4_ buckle, and α_1_ latch regions are indicated. (B) Close-up views of the *Sc* Ycg1–Brn1 interaction with DNA. (C) Schematic illustrations of the main contacts of *Sc* Ycg1 and Brn1 residues with the 18-bp DNAs in crystal forms I and II. Highly conserved residues are shown in bold, distant residues are shown in opaque. The length of the lines is proportional to the observed distances of H-bonds (solid lines) or ionic interactions (dotted lines). (D) Structural alignment of *Sc* Ycg1–Brn1 and *Sc* Ycg1–Brn1–DNA (crystal form I) using all C_α_ atoms (RMSD 1.95 Å over 820 C_α_). Arrows indicate conformational differences in the N-terminal shoulder region of Ycg1. Close-up views highlight changes of side chains in the Brn1 latch region. (E) Structural alignment of *Sc* Ycg1–Brn1–DNA crystal forms I and II DNA using all C_α_ atoms (RMSD 0.79 Å over 685 C_α_). Arrows indicate conformational differences in the DNA and in the C-terminal shoulder region of Ycg1. See also Figure S5.

To unambiguously determine the position and orientation of DNA bound to the Ycg1–Brn1 subcomplex, we solved the co-crystal structure of *Sc* Ycg1–Brn1 with 18-bp dsDNA to 2.98 Å resolution (Figure 4A and Table S1). As expected from the surface charge distribution and the results of the mutation experiments, we detected additional electron density corresponding to a DNA double helix within the Ycg1–Brn1 groove (Figure S5F). In the crystal, DNA duplexes pack via base-stacking interactions, with one copy of Ycg1–Brn1 contacting two-thirds of one DNA molecule and one-third of a symmetry-related DNA molecule (Figures 4A and S5F). The DNA helix is unwound by ∼65 degrees over 11 bp and bent by 7 degrees at one end, which results in an offset in the end-to-end stacking arrangement. This conformation is most likely necessary to compensate for the fact that the 18-bp DNA is slightly too long for the 1.5 helical turns required to produce the 2_1_-screw axis observed in the crystal and is not a consequence of binding to Ycg1–Brn1. Several conserved basic residues of both protein subunits, including those we had mutated (*Sc* Ycg1_K70, K71_, *Sc* Ycg1_K849_, *Sc* Brn1_K409, R411_, and *Sc* Brn1_K456_ corresponding to *Ct* Ycg1_K100, K101_, *Ct* Ycg1_K917_, *Ct* Brn1_R539, R541_, and *Ct* Brn1_K559_), interact with the phosphate backbone of both DNA strands (Figures 4B and 4C). Contacts with both backbones of the duplex and the absence of interactions with the nucleobases explain the lack of sequence specificity in DNA binding by the non-SMC subcomplex and preference for double- over single-stranded DNA (Piazza et al., 2014).

Comparison of free and DNA-bound structures shows that Ycg1 and Brn1 undergo fairly minor conformational changes upon DNA binding. In addition to a slight shift of the N- and C-terminal shoulders of Ycg1, the most notable difference upon DNA binding is the reorientation toward the double helix of residues within the α_1_ latch segment of Brn1, including two positively charged residues of the BC1 patch (*Sc* Brn1_K409_ and *Sc* Brn1_R411_; Figure 4D). The change in the conformation of the latch segment might either help to stabilize DNA binding or alter the affinity between the latch and the buckle. The latter hypothesis is consistent with the formation of hydrophobic contacts between a valine residue located between the two charged residues (*Sc* Brn1_V410_) and the buckle residues (*Sc* Ycg1_F808_ and *Sc* Brn1_W408_) in the DNA-bound structure (Figure 4D).

Whereas several basic residues of Ycg1 and Brn1 directly contact the DNA phosphate backbone, several other residues are oriented toward the DNA but are too far away to make direct electrostatic interactions (Figures 4B and 4C). As a result, the Ycg1–Brn1 groove might allow some degree of flexibility in accommodating the DNA double helix. This notion is further supported by a second crystal structure of Ycg1–Brn1 in complex with the same 18-bp DNA substrate, which crystallized with slightly different unit cell dimensions (Table S1). Compared to the first DNA co-structure, the C-terminal Ycg1 shoulder is slightly translocated (Figure 4E), and as a consequence, the contacts between this part of Ycg1–Brn1 and DNA differ considerably from the other crystal form (Figure 4C). The high elasticity in the conformation of the two Ycg1 shoulders and the low affinity measured for short DNA substrates *in vitro* (Figure 1C) raises the question of how condensin can nevertheless use the Ycg1–Brn1 groove to achieve a stable interaction with chromosomal DNA *in vivo*.

### The Brn1 Loop Encircles DNA

Even in the co-crystal structures of *Sc* Ycg1–Brn1 with DNA, 43 residues of the linker that connects the α_1_ and α_2_ segments of Brn1 remained disordered. The trajectories of the visible residues that flank the linker, however, indicate that the path of the connecting peptide leads over the bound DNA helix (Figure 4A). Since the length of this linker is considerably shorter in most other species (Figure S3B), we shortened the linker of *Sc* Brn1 by 27 residues to match the linker length of the human NCAPH kleisin subunit and co-crystallized the new construct with DNA (Table S1). The structure of this construct very closely resembles the previous *Sc* Ycg1–Brn1–DNA structures (Figure S5G). Although the shortened linker also remained disordered in this structure, the only conceivable way to span the distance of ∼36 Å between the two visible ends with the short linker is by running the connecting peptide over the DNA helix. As a consequence, the Brn1 kleisin subunit encircles the DNA double helix within the loop that is formed by the association of its N-terminal latch and C-terminal buckle segments (Figure 4A).

**Figure 5:**
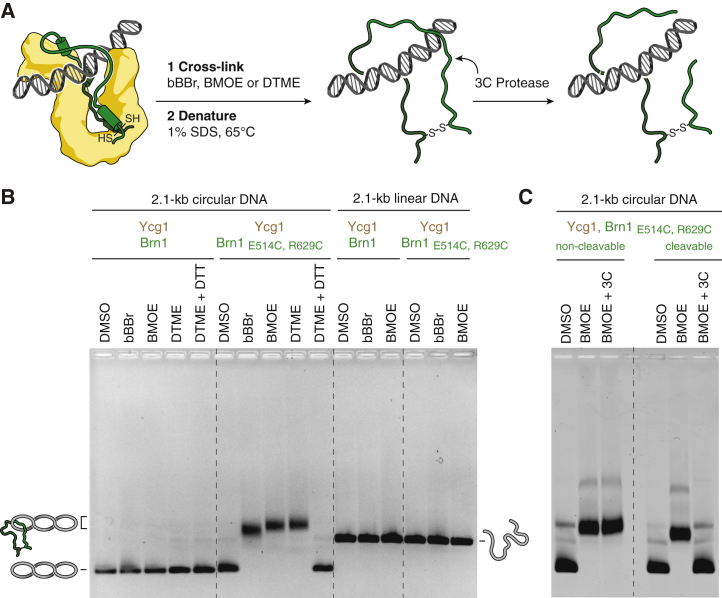
DNA Entrapment by the Kleisin Loop (A) Outline of the experimental setup to test whether the Brn1 loop encircles DNA. (B) Copurified *Ct* Ycg1_24–1006_–His_6_-TEV-Brn1_515–634_ subcomplexes without or with an additional cysteine pair engineered into the Brn1 latch and buckle regions (*Ct* Brn1_E514C, R629C_) were incubated with 2.1-kb circular or linear DNA substrates; DMSO solvent; or bBBr, BMOE, or DTME crosslinkers and denatured at 65°C in the presence of 1% SDS. Changes in DNA mobility were tested by EMSA and EtBr. (C) EMSA of a 2.1-kb circular DNA using copurified *Ct* Ycg1_24–1006_–His_6_-TEV-Brn1_515–634_ subcomplexes containing the Brn1_E514C, R629C_ cysteine pair and a target site for HRV-3C protease engineered following residue P549 in the *Ct* Brn1 linker region (cleavable) or no-protease site (non-cleavable) as described in (B). Following addition of DNA and incubation with DMSO solvent or BMOE crosslinker, samples were treated with HRV-3C protease (±3C) or buffer only. See also Figure S6.

**Figure S6:**
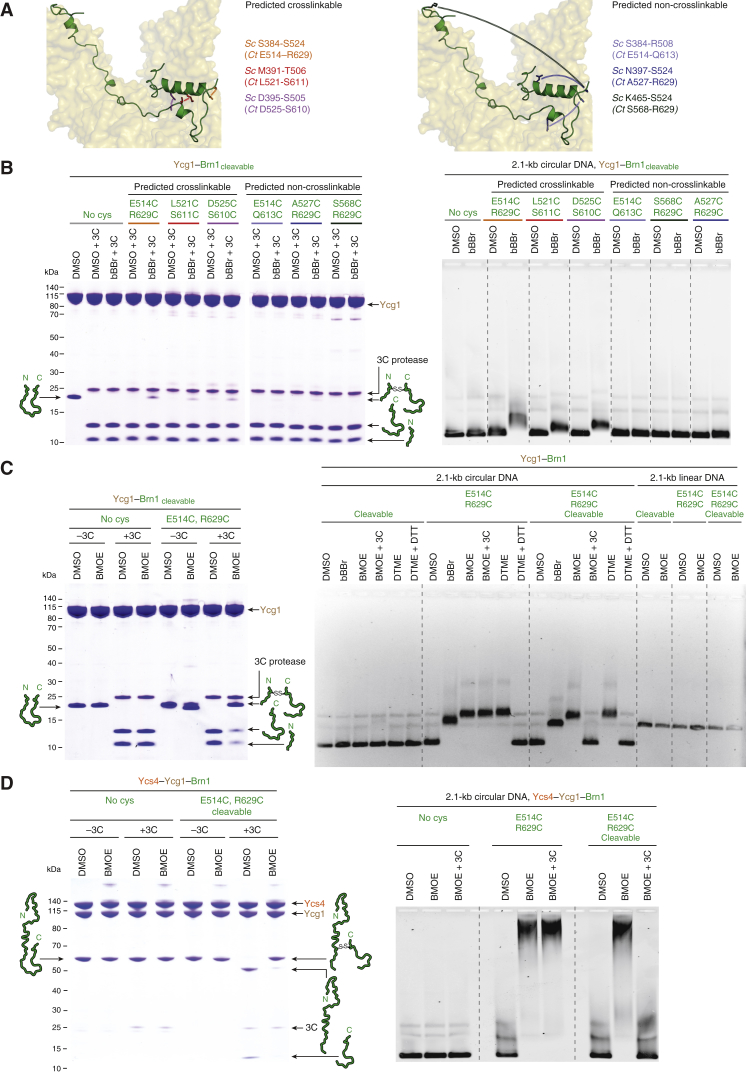
DNA Entrapment by the Kleisin Loop, Related to Figure 5 (A) Residue pairs in *Sc* Brn1 (green) latch and buckle segments in positions that should be crosslinkable (left panel, S384-S524, M391-T506, D395-S505) or in positions that should not be crosslinkable (right panel, S384-R508, N397-S524, K465-S524) when mutated to cysteine. Corresponding sequence homology pairs in *Ct* Brn1 (left panel, E514-R629, L521-S611, D525-S610; right panel, E514-Q613, A527-R629, S568-R629). (B) Analysis of copurified *Ct* Ycg1_24-1006_– His_6_-TEV-Brn1_515-634_ subcomplexes that either contain no additional cysteine residues (no cys) or additional cysteine pairs engineered into *Ct* Brn1 as in (listed in (A)) and a target sites for the 3C protease following Brn1 residue P549 (cleavable). Protein complexes were incubated with a 22-bp dsDNA before addition of DMSO solvent or bBBr crosslinker, followed by incubation with 3C protease (+3C) or buffer only (–3C), SDS-PAGE and Coomassie staining of two separate gels in parallel (left panel). EMSA of a 2.1-kb circular DNA with the same *Ct* Ycg1_24-1006_– His_6_-TEV-Brn1_515-634_ complexes detected by EtBr staining after incubation with DMSO or bBBr crosslinker and protein denaturation (right panel). (C) Analysis of copurified *Ct* Ycg1_24-1006_– His_6_-TEV-Brn1_515-634_ complexes without (no cys) or with an additional cysteine pair (Brn1_E514C, R629C_) engineered into the latch and buckle segments of Brn1 with (cleavable) or without a 3C protease site following Brn1 residue P549 as in (B), using bBBr, BMOE or DTME crosslinkers, followed by incubation with 3C protease (+3C), dithiothreitol (+DTT) or buffer only (see Figures 5B and 5C). (D) Analysis of copurified *Ct* Ycs4_3-1222_–Ycg1_24-1006_– His_6_-TEV-Brn1_225-634_ complexes without (no cys) or with an additional cysteine pair (Brn1_E514C, R629C_) engineered into the latch and buckle segments of Brn1 with (cleavable) or without a 3C protease site following Brn1 residue P549 as in (B), using BMOE crosslinker followed by incubation with 3C protease (+3C) or buffer only.

If this were indeed the case, then covalent connection between the contact points of the loop would create a circularized Brn1 peptide that should continue to entrap topologically restrained circular (but not linear) DNA even after protein denaturation (Figure 5A). We first engineered three pairs of cysteine residues into the Brn1 latch and buckle segments (*Ct* Brn1_E514C, R629C_, *Ct* Brn1_L521C, S611C_, and *Ct* Brn1_D525C, S610C_, which correspond to *Sc* Brn1_S384, S524_, *Sc* Brn1_M391, T506_, and *Sc* Brn1_D395, S505_) at positions that should be crosslinked with the thiol-reactive compound dibromobimane (bBBr; Figure S6A). In addition, we designed three pairs of cysteine residues in Brn1 that should be too far apart for crosslinking by bBBr (*Ct* Brn1_E514C, Q613C_, *Ct* Brn1_A527C, R629C_, and *Ct* Brn1_S568C, R629C_, which correspond to *Sc* Brn1_S384, R508_, *Sc* Brn1_N397, S524_, and *Sc* Brn1_K465, S524_). As expected, only the former three cysteine combinations produced a crosslink between the two ends of the Brn1 loop, which was detectable by SDS-PAGE after site-specific cleavage of the Brn1 linker region with human rhinovirus (HRV)-3C protease (Figure S6B). The crosslinking experiments hence confirm that, even in soluble Ycg1–Brn1 complexes, latch and buckle regions associate with each other as revealed by the crystal structures.

We then incubated Ycg1–Brn1 subcomplexes with circular DNA substrates before the addition of bBBr, denatured the proteins at 65°C with 1% SDS, and detected DNA by ethidium bromide (EtBr) staining after agarose gel electrophoresis. Addition of bBBr crosslinker, but not of DMSO solvent only, resulted in a marked reduction of the mobility of the DNA for the samples that contained crosslinkable Brn1 cysteine combinations, but not for samples without Brn1 cysteine pairs or with cysteine combinations that were too far apart to be crosslinked (Figure S6B). We selected one crosslinkable cysteine combination (*Ct* Brn1_E514C, R629C_) and repeated the experiment with two additional thiol-reactive compounds, bismaleimidoethane (BMOE) and dithiobismaleimidoethane (DTME), on linear and circular DNA substrates. Whereas crosslinking of *Ct* Ycg1–Brn1_E514C, R629C_ with BMOE or DTME, like bBBr, produced an upshift of the circular DNA, none of the crosslinkers produced an upshift of the linear DNA substrate (Figure 5B). These results suggest that the DNA upshift is due to the entrapment of the circular DNA species within the chemically circularized Brn1 loop, in contrast to linear DNA, which presumably can slide out of the Brn1 peptide loop.

Importantly, chemical cleavage of the covalent linkage between the Brn1 cysteine residues by reduction of the disulfide linker of DTME with dithiothreitol (DTT), as well as site-specific proteolytic cleavage of the Brn1 loop peptide backbone with HRV-3C protease after BMOE crosslinking, fully reverted the upshift of circular DNA substrates (Figures 5B, 5C, and S6C). Furthermore, addition of BMOE created a crosslink of the Brn1 loop and resulted in the formation of an SDS-resistant complex with circular DNA substrates even in the context of the *Ct* Ycs4–Ycg1–Brn1 ternary complex (Figure S6D). These experiments provide proof that the DNA bound within the Ycg1–Brn1 groove must be enclosed by the Brn1 loop.

### Association of Condensin with Mitotic Chromosomes Requires Brn1 Loop Closure around DNA

**Figure S7:**
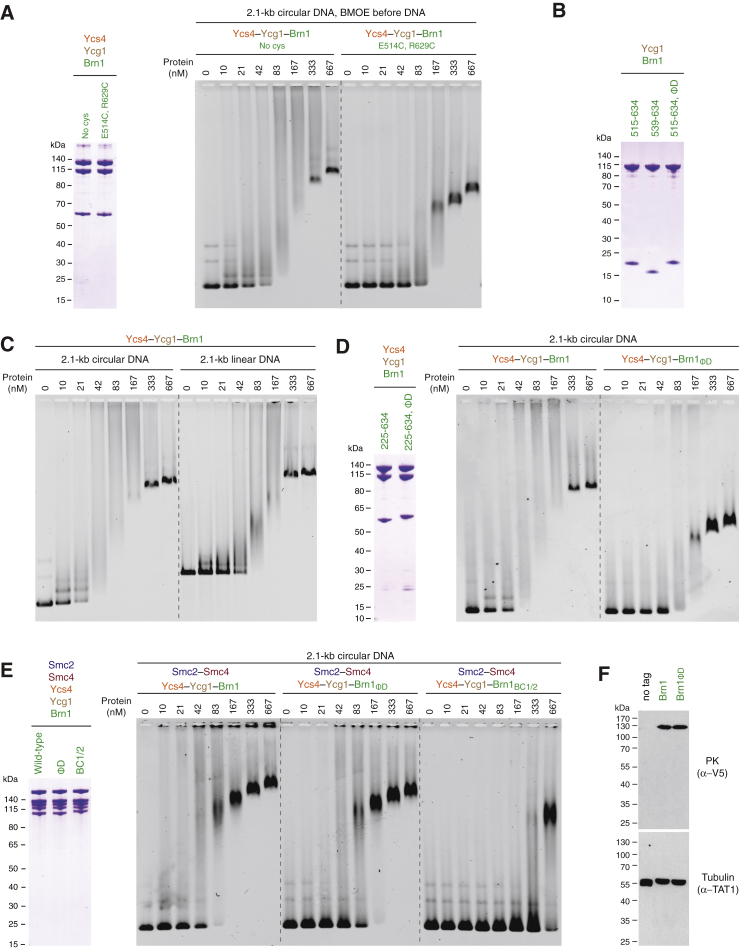
DNA Entrapment by the Brn1 Loop is Required for High-Affinity DNA Interaction, Related to Figure 6 (A) Analysis of copurified *Ct* Ycs4_3-1222_–Ycg1_24-1006_–His_6_-TEV-Brn1_225-634_ complexes without (no cys) or with an additional cysteine pair (Brn1_E514C, R629C_) engineered into the latch and buckle segments of Brn1 with a 3C protease site following Brn1 residue P549. Complexes were incubated with BMOE crosslinker before analysis by SDS-PAGE and Coomassie staining (left panel) or addition of a 2.1-kb circular DNA (10 nM), followed by EMSA and EtBr staining. Note the differences in the fraction of non-shifted DNA for each protein concentration. (B) *Ct* Ycg1_24-1006_– His_6_-TEV-Brn1 complexes used for EMSA in analyzed by SDS-PAGE and Coomassie staining (see Figures 6B and 6C). (C) EMSA analysis of the binding of the copurified *Ct* Ycs4_3-1222_–Ycg1_24-1006_–His_6_-TEV-Brn1_225-634_ complex to a 2.1-kb circular DNA (10 nM) or the same DNA linearized by restriction cleavage with *XmnI* (10 nM). (D) Analysis of copurified *Ct* Ycs4_3-1222_–Ycg1_24-1006_–His_6_-TEV-Brn1_225-634_ complexes as wild-type or Brn1_ΦD_ latch mutant versions (Brn1_L521D, F524D, W532D, W538D_) by SDS-PAGE and Coomassie staining (left panel). Protein complexes were used for EMSA with a 2.1-kb circular DNA (10 nM) substrate (right panel). (E) Analysis of *Sc* condensin holocomplexes (Smc2–Smc4-StrepII_3_–Ycs4–Ycg1–Brn1-His_12_-HA_3_) as wild-type, Brn1_ΦD_ latch mutant (Brn1_L521D, F524D, W532D, W538D_) or Brn1_BC1/2_ charge mutant (Brn1_K409D, R411D, K414D, K451D, K452D, K454D, K456D, K457D_) versions by SDS-PAGE and Coomassie staining (left panel). Protein complexes were used for EMSA with a 2.1-kb circular DNA (10 nM) substrate (right panel). (F) Brn1 expression levels of yeast strains C4237, C4239 and C4895 (see Figure 7C) analyzed by western blotting of whole cell lysates against the PK_6_ tag on Brn1 and α-tubulin as loading control.

Since latch and buckle segments are able to engage with each other even in the absence of DNA (Figure 2), the latch would need to disengage at least temporarily from the buckle to allow the entry of DNA into the Brn1 kleisin loop. If this were the case, locking latch and buckle segments by covalent crosslinking should prevent any subsequent DNA entrapment by the kleisin loop. We repeated the kleisin loop crosslinking experiment but, this time, crosslinked the *Ct* Brn1_E514C, R629C_ cysteine pair before adding DNA. This indeed reduced the affinity of the *Ct* Ycs4–Ycg1–Brn1 ternary complex for a circular DNA substrate (Figure S7A). DNA was still upshifted at higher protein concentrations, either because crosslinking had been incomplete or because the long flexible kleisin loop was able to assume an orientation that still allowed access to the Ycg1–Brn1 DNA binding groove even when its two ends had been covalently linked.

**Figure 6:**
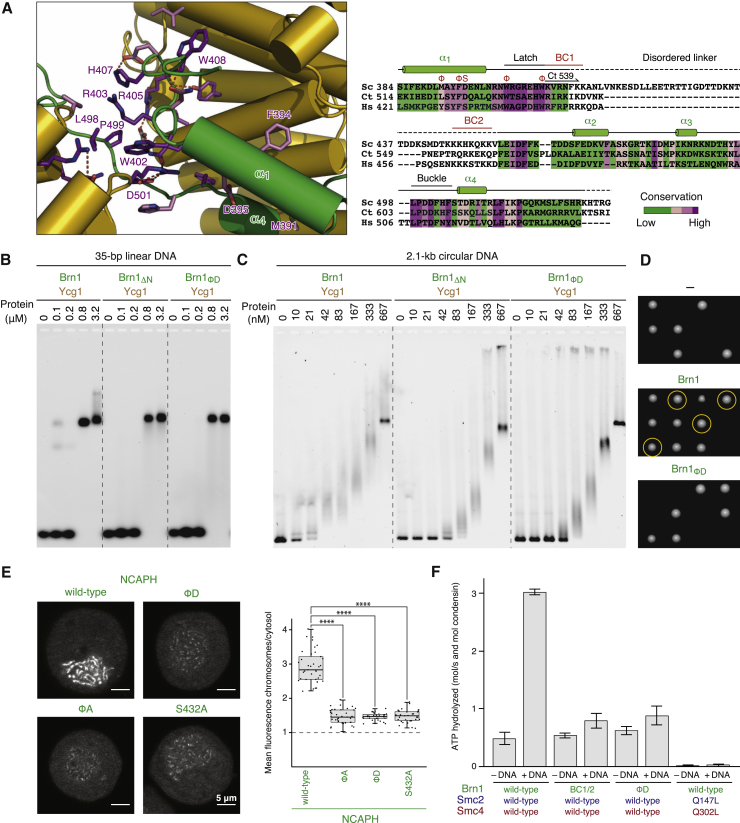
Kleisin Loop Closure Is Required for Condensin’s Loading onto Chromosomes and DNA-Dependent Stimulation of its ATPase (A) Close-up view of the *Sc* Brn1 latch-buckle interaction. Conserved Brn1 residues are marked in purple, relevant H-bonds are indicated (dotted lines). Sequence alignment of the Ycg1-interacting region of *Sc*, *Ct*, and *Hs* γ-kleisins. Secondary structure elements are highlighted based on the *Sc* Ycg1–Brn1 structure. Conserved latch and buckle regions, DNA binding site mutations (BC1 and BC2), mutations of hydrophobic latch residues (Φ), and the phosphorylated serine residue in *Hs* NCAPH_S432_ are highlighted. (B) EMSA of a 35-bp 6-FAM-labeled linear DNA (0.2 μM) with copurified wild-type (*Ct* Ycg1_24-1006_–His_6_-TEV-Brn1_515–634_), Brn1_ΔN_ truncated (*Ct* Ycg1_24–1006_–His_6_-TEV-Brn1_539–634_), and Brn1_ΦD_ latch mutant (*Ct* Ycg1_24–1006_–His_6_-TEV-Brn1_515–634, L521D, F524D, W532D, W538D_) subcomplexes detected by EtBr staining. (C) EMSA of a 2.1-kb circular DNA (10 nM) with the same proteins as described in (B) detected by EtBr staining. Note the differences in the fraction of non-shifted DNA. (D) Tetrad dissection of *BRN1*/*brn1Δ* diploid budding yeast cells (strains C4237, C4239, C4895) expressing ectopic copies of Brn1 wild-type or Brn1_ΦD_ (Brn1_M391D, F394D, W402D, W408D_) mutant versions of versions of Brn1-PK_6_. Images were recorded after three days at 25°C. Genetic marker analysis identifies *BRN1*_*x*_*, brn1Δ* cells (circles). (E) Representative example images of nocodazole-arrested HeLa cells expressing mCherry-tagged histone H2B and transiently transfected EGFP-tagged NCAPH as wild-type, latch mutant (ΦA: *Hs* NCAPH_Y428A, F431A, W439A, W445A_, ΦD: NCAPH_Y428D, F431D, W439D, W445D_), or non-phosphorylatable latch (*Hs* NCAPH_S432A_) versions. Scale bars: 5 μm. The graph plots ratios of chromosomal to cytosolic EGFP intensities. Horizontal lines indicate median, hinges indicate first and third quartiles, and whiskers extend to the highest or lowest point from the hinges within 1.5 times inter-quartile range, calculated from two independent experiments with a total of n = 37 (NCAPH), n = 37 (NCAPH_ΦA_), n = 31 (NCAPH_ΦD_), and n = 31 (NCAPH_S432A_) cells (p < 0.0001 by Student’s t test with Welch’s correction). (F) ATP hydrolysis by copurified *Sc* condensin holocomplexes (0.5 μM, Smc2–Smc4-StrepII_3_–Ycs4–Ycg1–Brn1-His_12_-HA_3_) containing wild-type, Brn1_BC1/2_ DNA binding (*Sc* Brn1_K409D, R411D, K414D, K451D, K452D, K454D, K456D, K457D_), Brn1_ΦD_ latch (*Sc* Brn1_M391D, F394D, W402D, W408D_), or ATPase deficient (*Sc* Smc2_Q147L_–Smc4_Q302L_) mutant versions with and without a 6.4-kb relaxed circular DNA at saturated ATP concentrations (5 mM). The plot shows mean ± SD from three independent experiments. See also Figure S7 and Table S2.

These findings suggest that the latch-buckle interaction is essential for condensin’s association with DNA. Notably, many residues of the Brn1 latch and buckle segments are remarkably conserved among condensin kleisin subunits (Figures 6A and S3B). To test the functional importance of the latch-buckle interaction, we shortened Brn1 to create a version that lacks the α_1_ latch segment (ΔN: *Ct* Brn1_539–634_) or mutated conserved hydrophobic residues within this region to negatively charged residues (ΦD: *Ct* Brn1_L521D, F524D, W532D, W538D_). Both modified versions of Brn1 still formed a complex with Ycg1 (Figure S7B), which confirms that the latch segment is not essential for the stable interaction between the two subunits.

We then compared binding of wild-type and mutant *Ct* Ycg1–Brn1 subcomplexes to short linear or long circular DNA substrates by EMSA (Figures 6B and 6C). All *Ct* Ycg1–Brn1 subcomplexes bound to the linear 35-bp DNA substrate with similar affinities (non-shifted DNA depleted at 800 nM protein; Figure 6B). This suggests that the kleisin loop is unable to stabilize binding to short linear DNA fragments, which might easily slide out of the loop. Wild-type *Ct* Ycg1–Brn1 subcomplexes shifted the 2.1-kb circular DNA at much lower protein concentrations (non-shifted DNA depleted at 21 nM; Figure 6C). The low micromolar value we measured by ITC for a short 25-bp duplex (Figure 1C) might hence considerably underestimate the affinity of Ycg1–Brn1 to DNA of physiologically relevant lengths, which can be topologically restrained by the kleisin loop. Importantly, shortening or mutation of the latch segment greatly reduced the affinity of *Ct* Ycg1–Brn1 subcomplexes for 2.1-kb circular DNA (non-shifted DNA depleted at 83 nM; Figure 6C). The *Ct* Ycs4–Ycg1–Brn1 ternary complex likewise bound a circular 2.1-kb DNA with higher affinity when compared to a linear DNA of the same size (Figure S7C). Mutation of the Brn1 latch region similarly increased the amounts of ternary complexes (Figure S7D) or even condensin holocomplexes (Figure S7E) required to shift the 2.1-kb circular DNA substrate, although the effect on DNA binding was less severe than mutation of the Brn1 BC1/2 patches.

Kleisin loop closure is furthermore important for chromosome association and function of condensin complexes *in vivo*, since the latch mutant version of Brn1 (ΦD: *Sc* Brn1_M391D, F394D, W402D, W408D_) failed to support proliferation in budding yeast (Figure 6D) despite being expressed at wild-type levels (Figure S7F). Mutation of the analogous residues in the human NCAPH kleisin subunit to either charged (ΦD: *Hs* NCAPH_Y428D, F431D, W439D, W445D_) or small hydrophobic side chains (ΦA: *Hs* NCAPH_Y428A, F431A, W439A, W445A_) dramatically reduced condensin association with mitotic chromosomes in cultured cells (Figure 6E). We conclude that while DNA seems to be able to access the basic Ycg1–Brn1 groove even without being encircled by the kleisin loop, only latch-mediated entrapment increases the affinity sufficiently to allow a stable interaction with chromosomes.

### DNA Entrapment by the Kleisin Loop Activates the Smc2–Smc4 ATPases

Addition of DNA had been reported to stimulate the ATPase activity of condensin holocomplexes, but not of Smc2–Smc4 dimers alone (Kimura and Hirano, 2000, Piazza et al., 2014, Stray and Lindsley, 2003). This implies that the DNA binding site in the Ycg1–Brn1 subcomplex might be able to act as a sensor that triggers activation of the Smc2–Smc4 ATPase activity. Whereas the presence of a 6.4-kb circular DNA enhanced the ATPase activity of *Sc* condensin holocomplexes containing wild-type Brn1 more than 5-fold, it had little effect on the ATPase rates measured for complexes containing *Sc* Brn1_BC1/2_ or *Sc* Brn1_ΦD_ mutant versions (Figure 6F). Brn1 loop closure around the DNA double helix is hence essential to activate ATP hydrolysis by the Smc2–Smc4 ATPase domains.

## Discussion

Although SMC protein complexes have been identified as the principal molecular machines that determine the three-dimensional organization of eukaryotic genomes, it had remained unclear how the complexes interact with their DNA substrates. Our structures reveal the formation of a composite DNA-binding groove by the HEAT-repeat and kleisin subunits and the entrapment of the DNA double helix by the kleisin peptide loop. This unconventional mode of DNA binding differs entirely from previously observed interactions with nucleic acids of other proteins that contain HEAT-repeat motifs (Cook et al., 2009, Okada et al., 2009, Rubinson et al., 2010). The facts that DNA contacts are made exclusively with the phosphate backbone and are conformationally adaptable provide the HEAT-repeat and kleisin subunits of condensin with the means to bind DNA independent of sequence or specific local structure, which contrasts sharply with the conventional concept of protein-DNA recognition (Rohs et al., 2010). This binding mode might target condensin to chromosomal positions where the double helix is freely accessible—for example, at transcriptionally active genes and other nucleosome-depleted regions (Robellet et al., 2017).

The absolute requirement for the Ycg1–Brn1 DNA-binding groove for the stable association of condensin with chromosomes in yeast and human cells suggests that it might play a direct role in loading condensin onto chromosomes and might thereby enable condensin to bypass the requirement for specific chromosomal loader proteins. Cohesin complexes, in contrast, rely on the function of a separate Scc2^Nipbl^–Scc4^Mau2^ dimer for their loading onto chromosomes (Ciosk et al., 2000). Since the architecture of the Scc2 HEAT-repeat solenoid very closely resembles the shape of the Ycg1 harp (Figure S4), it is tempting to speculate that both proteins might bind DNA in a similar manner.

The conformational flexibility in the contacts with DNA revealed by two Ycg1–Brn1–DNA crystal forms and the low binding affinity to short linear DNA fragments suggest that the positively charged groove alone can only weakly interact with DNA duplexes. Rapid dissociation of DNAs of physiologically relevant lengths bound in such a manner is only prevented by their entrapment within the kleisin peptide loop. The kleisin thereby acts analogous to a safety belt, akin to the way the safety belt of the spindle checkpoint protein Mad2 prevents dissociation of its protein ligands (Sironi et al., 2002). In this model, belt closure by association of latch and buckle regions anchors condensin to chromosomal DNA, which is consistent with the finding that mutation of conserved hydrophobic residues that are involved in the latch-buckle contact strongly reduces condensin binding to mitotic chromosomes. Since the safety belt cannot stabilize binding to short linear DNA substrates, it is conceivable that, even when entrapped by the kleisin loop, DNA double helices might be able to slide through the Ycg1–Brn1 groove, at least until they encounter a physical obstacle—for example, a nucleosome.

**Figure 7:**
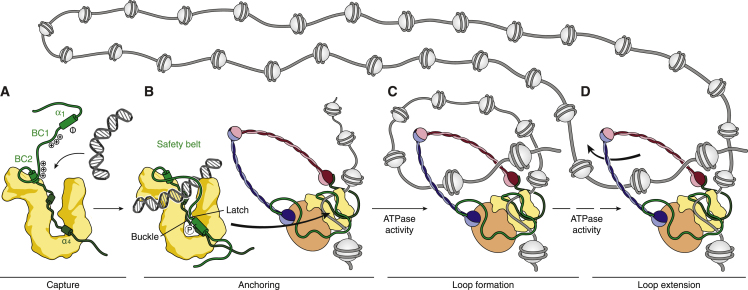
A Safety-Belt Mechanism Anchors Condensin to Chromosomes Model for a safety-belt mechanism of DNA binding by condensin complexes and condensin-mediated loop formation.

A corollary is that the safety belt needs to transiently open for DNA to enter the groove. Opening presumably occurs by dissociation of the kleisin latch, which would then expose the conserved positively charged BC1 and BC2 patches in the Brn1 linker region to assist in the capture of DNA double helices (Figure 7A). After DNA entry into the binding groove, association of Brn1 latch and buckle regions fastens the safety belt and results in stable DNA entrapment (Figure 7B). Notably, the α_1_ helix of *Hs* NCAPH contains a target motif for cyclin-dependent kinases (CDKs) and is phosphorylated during mitosis (Dephoure et al., 2008, Olsen et al., 2010). Mutation of the single serine residue within this motif, which is conserved among vertebrate species, to a non-phosphorylatable alanine residue almost completely abolishes condensin’s association with mitotic chromosomes (Figure 6E). The phosphorylated serine residue might interact with the conserved neighboring arginine and histidine residues of the buckle region (Figure 6A), which, in combination with the conformational changes of latch residues that we observe upon DNA binding (Figure 4D), presumably helps to lock the safety belt shut. Since we could not detect any sequence similarity between the α_1_ latch and loop regions of condensin and cohesin kleisin subunits, in contrast to sequence and structural similarities between the α_2_ to α_4_ segments (Figure S4), it is conceivable that the safety-belt mechanism might be specific to condensin.

The discovery of a DNA-binding activity in the Ycg1–Brn1 subcomplex does not rule out additional roles of these subunits. For example, the HEAT-repeat domains might promote the interaction between individual condensin complexes in a way as has been proposed for the interaction between cohesin complexes (Eng et al., 2015) or might drive the self-assembly of condensin complexes into a hydrogel-like network (Yoshimura and Hirano, 2016). DNA binding by Ycg1–Brn1 also does not exclude the existence of additional DNA binding sites in the condensin holocomplex. It is, for example, conceivable that Ycs4, even though it does not contribute significantly to the DNA-binding activity of condensin complexes *in vitro*, could do so *in vivo* (Kinoshita et al., 2015), possibly by acting as a sensor or modulator of the DNA-bound state. Other regions, including the Smc2–Smc4 hinge (Griese et al., 2010) or ATPase head domains, might form additional DNA interaction sites in the condensin holocomplex. These sites might only become accessible for DNA binding once the DNA double helix has been enclosed by the Brn1 kleisin loop and the Smc2–Smc4 ATPase has been activated.

We note that the presence of direct protein-DNA binding sites in condensin is not mutually exclusive with the topological entrapment of DNA within the Smc2–Smc4–Brn1 ring structure (Cuylen et al., 2011). Instead, it could function to anchor condensin complexes to chromosomes for their subsequent entrapment. In this scenario, activation of the Smc2–Smc4 ATPase activity, once DNA has been bound in the groove and the safety belt has been closed, results in a conformational change that opens the Smc2–Smc4–Brn1 ring for DNA entry (Figure 7C). Future studies will be needed to reveal whether DNA remains bound to the Ycg1–Brn1 groove after transport of the chromatin fiber into the ring. If so, holding on to the original binding site while condensin actively translocates away from this site (Figure 7D; Terekawa et al., 2017) would provide an elegant solution for how condensin complexes can generate the large-sized chromatin loops that are thought to constitute the basic organizational principle of higher-order chromosome architecture (Alipour and Marko, 2012, Nasmyth, 2001, Naumova et al., 2013).

## STAR★Methods

### Key Resources Table

**Table:** 

REAGENT or RESOURCE	SOURCE	IDENTIFIER
**Antibodies**		

Mouse monoclonal anti V5-tag (anti PK6-tag)	AbD Serotec (MCA1360)	RRID: AB_322378
Mouse monoclonal anti tubulin (TAT1)	Woods et al., 1989	N/A
Rabbit polyclonal anti *Sc* Ycg1	Piazza et al., 2014	N/A

**Bacterial and Virus Strains**		

*Escherichia coli* Rosetta (DE3) pLysS	Merck	Cat#70954

**Chemicals, Peptides, and Recombinant Proteins**		

Crosslinker dibromobimane (bBBr)	Sigma-Aldrich	Cat#34025
Crosslinker bismaleimidoethane (BMOE)	Thermo-Fisher	Cat#22323
Crosslinker disthiobismaleimidoethane (DTME)	Thermo-Fisher	Cat#22335
*C. thermophilum* Ycs4 (3-1222, N-terminal 6 × HIS-tag)	modified purification protocol from Piazza et al., 2014	N/A
*C. thermophilum* Ycg1 (24-1006, N-terminal 6 × HIS-tag)	modified purification protocol from Piazza et al., 2014	N/A
*C. thermophilum* Brn1_336-714_ (336-714, N-terminal GST-tag)	This work	N/A
*C. thermophilum* Brn1_515-634_ (515-634, N-terminal 6 × HIS-tag) in complex with Ycg1 (24-1006)	This work	N/A
*C. thermophilum* Brn1_515-634, BC1_ (515-634, R539D, R541D, K542D, K544D, N-terminal 6 × HIS-tag) in complex with Ycg1 (24-1006)	This work	N/A
*C. thermophilum* Brn1_515-634, BC2_ (515-634, R554D, R556D, K557D, K559D, N-terminal 6 × HIS-tag) in complex with Ycg1 (24-1006)	This work	N/A
*C. thermophilum* Brn1_515-634, BC1/2_ (515-634, R539D, R541D, K542D, K544D, R554D, R556D, K557D, K559D, N-terminal 6 × HIS-tag) in complex with Ycg1 (24-1006)	This work	N/A
*C. thermophilum* Brn1_515-634_ (515-634, N-terminal 6 × HIS-tag) in complex with Ycg1_YC1_ (24-1006, K100D, K101D)	This work	N/A
*C. thermophilum* Brn1_515-634_ (515-634, N-terminal 6 × HIS-tag) in complex with Ycg1_YC2_ (24-1006, K916D, K917D)	This work	N/A
*C. thermophilum* Brn1_515-634_ (515-634, N-terminal 6 × HIS-tag) in complex with Ycg1_YC1/2_ (24-1006, K100D, K101D, K916D, K917D)	This work	N/A
*C. thermophilum* Brn1_225-512_ (225-512, N-terminal 6 × HIS-tag) in complex with Ycs4 (3-1222)	This work	N/A
*C. thermophilum* Brn1_539-634_ (539-634, N-terminal 6 × HIS-tag) in complex with Ycg1 (24-1006)	This work	N/A
*C. thermophilum* Brn1_549-634_ (549-634, N-terminal 6 × HIS-tag) in complex with Ycg1 (24-1006)	This work	N/A
*C. thermophilum* Brn1_572-634_ (572-634, N-terminal 6 × HIS-tag) in complex with Ycg1 (24-1006)	This work	N/A
*C. thermophilum* Brn1_515-601_ (515-601, N-terminal 6 × HIS-tag) in complex with Ycg1 (24-1006)	This work	N/A
*C. thermophilum* Brn1_515-634_ (515-634, N-terminal 6 × HIS-tag) in complex with Ycg1_78-1006_ (78-1006)	This work	N/A
*C. thermophilum* Brn1_515-634_ (515-634, N-terminal 6 × HIS-tag) in complex with Ycg1_24-823_ (24-823)	This work	N/A
*C. thermophilum* Brn1_515-634_ (515-634, N-terminal 6 × HIS-tag) in complex with Ycg1_24-883_ (24-883)	This work	N/A
*C. thermophilum* Brn1_515-634_ (515-634, N-terminal 6 × HIS-tag) in complex with Ycg1_24-934_ (24-934)	This work	N/A
*C. thermophilum* Brn1_515-634_ (515-634, N-terminal 6 × HIS-tag) in complex with Ycg1_24-982_ (24-982)	This work	N/A
*C. thermophilum* Brn1_515-634, E514C-R629C_ (515-634, E514C, R629C, N-terminal 6 × HIS-tag) in complex with Ycg1 (24-1006)	This work	N/A
*C. thermophilum* Brn1_515-634, E514C-R629C cleavable_ (515-634, E514C, R629C, N-terminal 6 × HIS-tag, HRV3C cleavage site inserted after P549) in complex with Ycg1 (24-1006)	This work	N/A
*C. thermophilum* Brn1_515-634, L521C-S611C cleavable_ (515-634, L521C, S611C, N-terminal 6 × HIS-tag, HRV3C cleavage site inserted after P549) in complex with Ycg1 (24-1006)	This work	N/A
*C. thermophilum* Brn1_515-634, D525C-S610C cleavable_ (515-634, D525C, S610C, N-terminal 6 × HIS-tag, HRV3C cleavage site inserted after P549) in complex with Ycg1 (24-1006)	This work	N/A
*C. thermophilum* Brn1_515-634, E514C-Q613C cleavable_ (515-634, E514C, Q613C, N-terminal 6 × HIS-tag, HRV3C cleavage site inserted after P549) in complex with Ycg1 (24-1006)	This work	N/A
*C. thermophilum* Brn1_515-634, A527C-R629C cleavable_ (515-634, A527C, R629C, N-terminal 6 × HIS-tag, HRV3C cleavage site inserted after P549) in complex with Ycg1 (24-1006)	This work	N/A
*C. thermophilum* Brn1_515-634, S568C-R629C cleavable_ (515-634, S568C, R629C, N-terminal 6 × HIS-tag, HRV3C cleavage site inserted after P549) in complex with Ycg1 (24-1006)	This work	N/A
*C. thermophilum* Brn1_515-634, cleavable_ (515-634, N-terminal 6 × HIS-tag, HRV3C cleavage site inserted after P549) in complex with Ycg1 (24-1006)	This work	N/A
*C. thermophilum* Brn1_225-634_ (225-634, N-terminal 6 × HIS-tag) in complex with Ycg1 (24-1006) and Ycs4 (3-1222)	This work	N/A
*C. thermophilum* Brn1_225-634_ (225-634, N-terminal 6 × HIS-tag) in complex with Ycg1_YC1+YC2_ (24-1006, K100D, K101D, K916D, K917D) and Ycs4 (3-1222)	This work	N/A
*C. thermophilum* Brn1_225-634 BC1/2_ (225-634, R539D, R541D, K542D, K544D, R554D, R556D, K557D, K559D, N-terminal 6 × HIS-tag) in complex with Ycg1 (24-1006) and Ycs4 (3-1222)	This work	N/A
*C. thermophilum* Brn1_225-634, E514C-R629C_ (225-634, E514C, R629C, N-terminal 6 × HIS-tag) in complex with Ycg1 (24-1006) and Ycs4 (3-1222)	This work	N/A
*C. thermophilum* Brn1_225-634, E514C-R629C cleavable_ (225-634, E514C, R629C, N-terminal 6 × HIS-tag, HRV3C cleavage site inserted after P549) in complex with Ycg1 (24-1006) and Ycs4 (3-1222)	This work	N/A
*C. thermophilum* Brn1_225-634_, _ΦD_ (225-634, L521D, F524D, W532D, W538D, N-terminal 6 × HIS-tag) in complex with Ycg1 (24-1006) and Ycs4 (3-1222)	This work	N/A
*C. thermophilum* Brn1_515-634_, _ΦD_ (515-634, L521D, F524D, W532D, W538D, N-terminal 6 × HIS-tag) in complex with Ycg1 (24-1006)	This work	N/A
*S. cerevisiae* Smc2 (full length) in complex with Smc4 (full length, C-terminal 3 × StrepII-tag), Brn1 (full length, C-terminal 12 × HIS-tag 3 × HA-tag), Ycg1 (full length) and Ycs4 (full length)	Terekawa et al., 2017	N/A
*S. cerevisiae* Smc2 (full length) in complex with Smc4 (full length, C-terminal 3 × StrepII-tag), Brn1 (full length, C-terminal 12 × HIS-tag 3 × HA-tag) and Ycs4 (full length)	This work	N/A
*S. cerevisiae* Smc2 (full length) in complex with Smc4 (full length, C-terminal 3 × StrepII-tag), Brn1_BC1/2_ (full length, K409D, R411D, K414D, K451D, K452D, K454D, K456D, K457D, C-terminal 12 × HIS-tag 3 × HA-tag), Ycg1 (full length) and Ycs4 (full length)	This work	N/A
*S. cerevisiae* Smc2 (full length) in complex with Smc4 (full length, C-terminal 3 × StrepII-tag), Brn1_ΦD_ (full length, M391D, F394D, W402D, W408D, C-terminal 12 × HIS-tag 3 × HA-tag), Ycg1 (full length) and Ycs4 (full length)	This work	N/A
*S. cerevisiae* Smc2_Q147L_ (full length) in complex with Smc4_Q302L_ (full length, C-terminal 3 × StrepII-tag), Brn1 (full length, C-terminal 12 × HIS-tag 3 × HA-tag), Ycg1 (full length) and Ycs4 (full length)	Terekawa et al., 2017	N/A
*S. cerevisiae* Brn1 (384-529) Ycg1 (6-498, 556-932)	This work	N/A
*S. cerevisiae* Brn1_short kleisin loop_ (384-417, 445-529) Ycg1 (6-498, 556-932)	This work	N/A
*S. pombe* Cnd2 (416-544) Cnd3 (1-438, 474-823)	This work	N/A

**Deposited Data**		

*S. pombe* Cnd3–Cnd2	This work	PDB: 5OQR
*S. cerevisiae* Ycg1–Brn1	This work	PDB: 5OQQ
*S. cerevisiae* Ycg1–Brn1–DNA (I)	This work	PDB: 5OQP
*S. cerevisiae* Ycg1–Brn1–DNA (II)	This work	PDB: 5OQO
*S. cerevisiae* Ycg1–Brn1–DNA (short kleisin loop)	This work	PDB: 5OQN

**Experimental Models: Cell Lines**		

HeLa Kyoto H2B-mCherry	Neumann et al., 2010	N/A

**Experimental Models: Organisms/Strains**		

*S. cerevisiae* C4237 (*MATa/α, ade2-1, trp1-1, can1-100, leu2-3,112, GAL, psi+, brn1::HIS3/BRN1, ura3::empty-vector::URA3/ura3*)	This work	N/A
*S. cerevisiae* C4239 (*MATa/α ade2-1, trp1-1, can1-100, leu2-3,112, GAL, psi+, brn1::HIS3/BRN1, ura3::BRN1-PK*_*6*_*::URA3/ura3*)	This work	N/A
*S. cerevisiae* C4257 (*MATa/α ade2-1, trp1-1, can1-100, leu2-3,112, GAL, psi+, brn1::HIS3/BRN1, ura3::brn1(K409D, R411D, K414D)-PK*_*6*_*::URA3/ura3*)	This work	N/A
*S. cerevisiae* C4259 (*MATa/α ade2-1, trp1-1, can1-100, leu2-3,112, GAL, psi+, brn1::HIS3/BRN1, ura3::brn1(K451D, K452D, K454D, K456D, K457D)-PK*_*6*_*::URA3/ura3*)	This work	N/A
*S. cerevisiae* C4261 (*MATa/α ade2-1, trp1-1, can1-100, leu2-3,112, GAL, psi+, brn1::HIS3/BRN1, ura3::brn1(K409D, R411D, K414D, K451D, K452D, K454D, K456D, K457D)-PK*_*6*_*::URA3/ura3*)	This work	N/A
*S. cerevisiae* C4491 (*MATa, lys2::pGAL1 GAL4::LYS2, pep4::HIS3, bar1::hisG, [2micron pGAL7 SMC4-StrepII*_*3*_*, pGAL10 SMC2, pGAL1 BRN1-His*_*12*_*-HA*_*3*_*, TRP1], [2micron pGAL1 YCG1, pGAL10 YCS4, URA3]*)	Terekawa et al., 2017	N/A
*S. cerevisiae* C4493 (*MATa, lys2::pGAL1 GAL4::LYS2, pep4::HIS3, bar1::hisG, [2micron pGAL7 SMC4-StrepII*_*3*_*, pGAL10 SMC2, pGAL1 BRN1-His*_*12*_*-HA*_*3*_*, TRP1], [2micron pGAL10 YCS4, URA3]*)	This work	N/A
*S. cerevisiae* C4516 (*MATa, lys2::pGAL1 GAL4::LYS2, pep4::HIS3, bar1::hisG, [2micron pGAL7 SMC4-StrepII*_*3*_*, pGAL10 SMC2, pGAL1 BRN1(K409D, R411D, K414D, K451D, K452D, K456D, K457D)-His*_*12*_*-HA*_*3*_*, TRP1], [2micron pGAL1 YCG1, pGAL10 YCS4, URA3]*)	This work	N/A
*S. cerevisiae* C4724 (*MATa, lys2::pGAL1 GAL4::LYS2, pep4::HIS3, bar1::hisG, [2micron pGAL7 SMC4(Q302L)- StrepII*_*3*_*, pGAL10 SMC2(Q147L), pGAL1 BRN1-His*_*12*_*-HA*_*3*_*, TRP1], [2micron pGAL1 YCG1, pGAL10 YCS4, URA3]*)	Terekawa et al., 2017	N/A
*S. cerevisiae* C4895 (*MATa/α ade2-1, trp1-1, can1-100, leu2-3,112, GAL, psi+, brn1::HIS3/BRN1, ura3::brn1(M391D, F394D, W402D, W408D)-PK*_*6*_*::URA3/ura3*)	This work	N/A
*S. cerevisiae* C5037 (*MATa, lys2::pGAL1 GAL4::LYS2, pep4::HIS3, bar1::hisG, [2micron pGAL7 SMC4-StrepII*_*3*_*, pGAL10 SMC2, pGAL1 BRN1(M391D, F394D, W402D, W408D)-His*_*12*_*-HA*_*3*_*, TRP1], [2micron pGAL1 YCG1, pGAL10 YCS4, URA3]*)	This work	N/A

**Oligonucleotides**		

EMSA 35-mer template forward strand 5′-6-FAM-CCTATAGTGAGTCGTTCGATATTACAATTC ACTGG-3′	This work/modified from Piazza et al., 2014	N/A
EMSA 35-mer template reverse strand 5′-CCAGTGAATTGTAATATCGAACGACTCACTATAG G-3′	This work/modified from Piazza et al., 2014	N/A
ITC 25-mer annealed forward strand 5′-CCTATAGTGAGTCACAATTCACTGG-3′	This work/modified from Piazza et al., 2014	N/A
ITC 25-mer annealed reverse strand 5′-CCAGTGAATTGTGACTCACTATAGG-3′	This work/modified from Piazza et al., 2014	N/A
Crystallization 18-mer annealed palindromic dsDNA 5′-GATGTGTAGCTACACATC-3′	This work	N/A
22-mer annealed palindromic dsDNA 5′-GATTCGTGTAGCTACACGAATC-3′	Seifert et al., 2016	N/A
qPCR CEN4 (SC-77) forward primer 5′-TGGTGTGGAAGTCCTAATATCG-3′	Cuylen et al., 2011	N/A
qPCR CEN4 (SC-78) reverse primer 5′-TGCATGATCAAAAGGCTCAA-3′	Cuylen et al., 2011	N/A
qPCR rDNA (SC-41) forward primer 5′-TTTCTGCCTTTTTCGGTGAC-3′	Cuylen et al., 2011	N/A
qPCR CEN4 (SC-42) reverse primer 5′-TGGCATGGATTTCCCTTTAG-3′	Cuylen et al., 2011	N/A

**Recombinant DNA**		

Plasmid pGEX6PI-Brn1_336-714_ (N-terminal GST-tag-HRV3C-cleavage-site, residues 336-714 of *C. thermophilum* Brn1)	This work	N/A
Plasmid pETMCN-Ycs4 (N-terminal 6 × HIS-tag-TEV-cleavage-site, residues 3-1222 of *C. thermophilum* Ycs4)	This work	N/A
Plasmid pETMCN-Ycg1 (N-terminal 6 × HIS-tag-TEV-cleavage-site, residues 24-1006 of *C. thermophilum* Ycg1)	This work	N/A
Plasmid pETMCN-Brn1_515-634_-Ycg1 (N-terminal 6 × HIS-tag-TEV-cleavage-site, residues 515-634 of *C. thermophilum* Brn1 and residues 24-1006 of untagged *C. thermophilum* Ycg1)	This work	N/A
Plasmid pETMCN-Brn1_515-634_, _BC1_ -Ycg1 (N-terminal 6 × HIS-tag-TEV-cleavage-site, residues 515-634 with mutated residues R539D, R541D, K542D, K544D of *C. thermophilum* Brn1 and residues 24-1006 of untagged *C. thermophilum* Ycg1)	This work	N/A
Plasmid pETMCN-Brn1_515-634_, _BC2_ -Ycg1 (N-terminal 6 × HIS-tag-TEV-cleavage-site, residues 515-634 with mutated residues R554D, R556D, K557D, K559D of *C. thermophilum* Brn1 and residues 24-1006 of untagged *C. thermophilum* Ycg1)	This work	N/A
Plasmid pETMCN-Brn1_515-634_, _BC1/2_ -Ycg1 (N-terminal 6 × HIS-tag-TEV-cleavage-site, residues 515-634 with mutated residues R539D, R541D, K542D, K544D, R554D, R556D, K557D, K559D of *C. thermophilum* Brn1 and residues 24-1006 of untagged *C. thermophilum* Ycg1)	This work	N/A
Plasmid pETMCN-Brn1_515-634_ -Ycg1_YC1_ (N-terminal 6 × HIS-tag-TEV-cleavage-site, residues 515-634 of *C. thermophilum* Brn1 and residues 24-1006 with mutated residues K100D, K101D of untagged *C. thermophilum* Ycg1)	This work	N/A
Plasmid pETMCN-Brn1_515-634_ -Ycg1_YC2_ (N-terminal 6 × HIS-tag-TEV-cleavage-site, residues 515-634 of *C. thermophilum* Brn1 and residues 24-1006 with mutated residues K916D, K917D of untagged *C. thermophilum* Ycg1)	This work	N/A
Plasmid pETMCN-Brn1_515-634_ -Ycg1_YC1+YC2_ (N-terminal 6 × HIS-tag-TEV-cleavage-site, residues 515-634 of *C. thermophilum* Brn1 and residues 24-1006 with mutated residues K100D, K101D, K916D, K917D of untagged *C. thermophilum* Ycg1)	This work	N/A
Plasmid pETMCN-Brn1_225-512_-Ycs4 (N-terminal 6 × HIS-tag-TEV-cleavage-site, residues 225-512 of *C. thermophilum* Brn1 and residues 3-1222 of untagged *C. thermophilum* Ycs4)	This work	N/A
Plasmid pETMCN-Brn1_539-634_-Ycg1 (N-terminal 6 × HIS-tag-TEV-cleavage-site, residues 539-634 of *C. thermophilum* Brn1 and residues 24-1006 of untagged *C. thermophilum* Ycg1)	This work	N/A
Plasmid pETMCN-Brn1_549-634_-Ycg1 (N-terminal 6 × HIS-tag-TEV-cleavage-site, residues 549-634 of *C. thermophilum* Brn1 and residues 24-1006 of untagged *C. thermophilum* Ycg1)	This work	N/A
Plasmid pETMCN-Brn1_572-634_-Ycg1 (N-terminal 6 × HIS-tag-TEV-cleavage-site, residues 572-634 of *C. thermophilum* Brn1 and residues 24-1006 of untagged *C. thermophilum* Ycg1)	This work	N/A
Plasmid pETMCN-Brn1_515-601_-Ycg1 (N-terminal 6 × HIS-tag-TEV-cleavage-site, residues 515-601 of *C. thermophilum* Brn1 and residues 24-1006 of untagged *C. thermophilum* Ycg1)	This work	N/A
Plasmid pETMCN-Brn1_515-634_-Ycg1_78-1006_ (N-terminal 6 × HIS-tag-TEV-cleavage-site, residues 515-634 of *C. thermophilum* Brn1 and residues 78-1006 of untagged *C. thermophilum* Ycg1)	This work	N/A
Plasmid pETMCN-Brn1_515-634_-Ycg1_24-823_ (N-terminal 6 × HIS-tag-TEV-cleavage-site, residues 515-634 of *C. thermophilum* Brn1 and residues 24-823 of untagged *C. thermophilum* Ycg1)	This work	N/A
Plasmid pETMCN-Brn1_515-634_-Ycg1_24-883_ (N-terminal 6 × HIS-tag-TEV-cleavage-site, residues 515-634 of *C. thermophilum* Brn1 and residues 24-883 of untagged *C. thermophilum* Ycg1)	This work	N/A
Plasmid pETMCN-Brn1_515-634_-Ycg1_24-934_ (N-terminal 6 × HIS-tag-TEV-cleavage-site, residues 515-634 of *C. thermophilum* Brn1 and residues 24-934 of untagged *C. thermophilum* Ycg1)	This work	N/A
Plasmid pETMCN-Brn1_515-634_-Ycg1_24-982_ (N-terminal 6 × HIS-tag-TEV-cleavage-site, residues 515-634 of *C. thermophilum* Brn1 and residues 24-982 of untagged *C. thermophilum* Ycg1)	This work	N/A
Plasmid pETMCN-Brn1_515-634, E514C-R629C cleavable_-Ycg1 (N-terminal 6 × HIS-tag-TEV-cleavage-site, residues 515-634 with mutated residues E514C, R629C and HRV3C cleavage site inserted after P549 of *C. thermophilum* Brn1 and residues 24-1006 of untagged *C. thermophilum* Ycg1)	This work	N/A
Plasmid pETMCN-Brn1_515-634, L521C-S611C cleavable_-Ycg1 (N-terminal 6 × HIS-tag-TEV-cleavage-site, residues 515-634 with mutated residues L521C, S611C and HRV3C cleavage site inserted after P549 of *C. thermophilum* Brn1 and residues 24-1006 of untagged *C. thermophilum* Ycg1)	This work	N/A
Plasmid pETMCN-Brn1_515-634, D525C-S610C cleavable_-Ycg1 (N-terminal 6 × HIS-tag-TEV-cleavage-site, residues 515-634 with mutated residues D525C, S610C and HRV3C cleavage site inserted after P549 of *C. thermophilum* Brn1 and residues 24-1006 of untagged *C. thermophilum* Ycg1)	This work	N/A
Plasmid pETMCN-Brn1_515-634, E514C-Q613C cleavable_-Ycg1 (N-terminal 6 × HIS-tag-TEV-cleavage-site, residues 515-634 with mutated residues E514C, Q613C and HRV3C cleavage site inserted after P549 of *C. thermophilum* Brn1 and residues 24-1006 of untagged *C. thermophilum* Ycg1)	This work	N/A
Plasmid pETMCN-Brn1_515-634, A527C-R629C cleavable_-Ycg1 (N-terminal 6 × HIS-tag-TEV-cleavage-site, residues 515-634 with mutated residues A527C, R629C and HRV3C cleavage site inserted after P549 of *C. thermophilum* Brn1 and residues 24-1006 of untagged *C. thermophilum* Ycg1)	This work	N/A
Plasmid pETMCN-Brn1_515-634, S568C-R629C cleavable_-Ycg1 (N-terminal 6 × HIS-tag-TEV-cleavage-site, residues 515-634 with mutated residues S568C, R629C and HRV3C cleavage site inserted after P549 of *C. thermophilum* Brn1 and residues 24-1006 of untagged *C. thermophilum* Ycg1)	This work	N/A
Plasmid pETMCN-Brn1_515-634, E514C-R629C_-Ycg1 (N-terminal 6 × HIS-tag-TEV-cleavage-site, residues 515-634 with mutated residues E514C, R629C of *C. thermophilum* Brn1 and residues 24-1006 of untagged *C. thermophilum* Ycg1)	This work	N/A
Plasmid pETMCN-Brn1_515-634, cleavable_-Ycg1 (N-terminal 6 × HIS-tag-TEV-cleavage-site, residues 515-634 with HRV3C cleavage site inserted after P549 of *C. thermophilum* Brn1 and residues 24-1006 of untagged *C. thermophilum* Ycg1)	This work	N/A
Plasmid pETMCN-Brn1_225-634_-Ycg1 (N-terminal 6 × HIS-tag-TEV-cleavage-site, residues 225-634 of *C. thermophilum* Brn1 and residues 24-1006 of untagged *C. thermophilum* Ycg1)	This work	N/A
Plasmid pETMCN-Brn1_225-634_-Ycg1_YC1+YC2_ (N-terminal 6 × HIS-tag-TEV-cleavage-site, residues 225-634 of *C. thermophilum* Brn1 and residues 24-1006 with mutated residues K100D, K101D, K916D, K917D of untagged *C. thermophilum* Ycg1)	This work	N/A
Plasmid pETMCN-Brn1_225-634, BC1/2_-Ycg1 (N-terminal 6 × HIS-tag-TEV-cleavage-site, residues 225-634 with mutated residues R539D, R541D, K542D, K544D, R554D, R556D, K557D, K559D of *C. thermophilum* Brn1 and residues 24-1006 of untagged *C. thermophilum* Ycg1)	This work	N/A
Plasmid pETMCN-Brn1_225-634, E514C-R629C_-Ycg1 (N-terminal 6 × HIS-tag-TEV-cleavage-site, residues 225-634 with mutated residues E514C, R629C of *C. thermophilum* Brn1 and residues 24-1006 of untagged *C. thermophilum* Ycg1)	This work	N/A
Plasmid pETMCN-Brn1_225-634, E514C-R629C cleavable_-Ycg1 (N-terminal 6 × HIS-tag-TEV-cleavage-site, residues 225-634 with mutated residues E514C, R629C and HRV3C cleavage site inserted after P549 of *C. thermophilum* Brn1 and residues 24-1006 of untagged *C. thermophilum* Ycg1)	This work	N/A
Plasmid pETMCN-Brn1_225-634_, _ΦD_ -Ycg1 (N-terminal 6 × HIS-tag-TEV-cleavage-site, residues 225-634 with mutated residues L521D, F524D, W532D, W538D of *C. thermophilum* Brn1 and residues 24-1006 of untagged *C. thermophilum* Ycg1)	This work	N/A
Plasmid pETMCN-Brn1_515-634_, _ΦD_ -Ycg1 (N-terminal 6 × HIS-tag-TEV-cleavage-site, residues 515-634 with mutated residues L521D, F524D, W532D, W538D of *C. thermophilum* Brn1 and residues 24-1006 of untagged *C. thermophilum* Ycg1)	This work	N/A
Plasmid 2micron pGAL7 SMC4-StrepII_3_ (C-terminal 3 × StrepII-tag of *S. cerevisiae* full length Smc4), pGAL10 SMC2 (*S. cerevisiae* full length Smc2), pGAL1 BRN1-His_12_-HA_3_ (C-terminal 12 × HIS-3 × HA-tag of *S. cerevisiae* full length Brn1), TRP1	St-Pierre et al., 2009	N/A
Plasmid 2micron pGAL7 SMC4-StrepII_3_ (C-terminal 3 × StrepII-tag with mutated residue Q302L of *S. cerevisiae* full length Smc4), pGAL10 SMC2 (with mutated residue Q147L of *S. cerevisiae* full length Smc2), pGAL1 BRN1-His_12_-HA_3_ (C-terminal 12 × HIS-3 × HA-tag of *S. cerevisiae* full length Brn1), TRP1	Terekawa et al., 2017	N/A
Plasmid 2micron pGAL1 YCG1 (*S. cerevisiae* full length Ycg1), pGAL10 YCS4 (*S. cerevisiae* full length Ycs4), URA3	Terekawa et al., 2017	N/A
Plasmid 2micron pGAL7 SMC4-StrepII_3_ (C-terminal 3 × StrepII-tag of *S. cerevisiae* full length Smc4), pGAL10 SMC2 (*S. cerevisiae* full length Smc2), pGAL1 BRN1-His_12_-HA_3_ (C-terminal 12 × HIS-3 × HA-tag with mutated residues K409D, R411D, K414D, K451D, K452D, K454D, K456D, K457D of *S. cerevisiae* full length Brn1), TRP1	This work	N/A
Plasmid 2micron pGAL7 SMC4-StrepII_3_ (C-terminal 3 × StrepII-tag of *S. cerevisiae* full length Smc4), pGAL10 SMC2 (*S. cerevisiae* full length Smc2), pGAL1 BRN1-His_12_-HA_3_ (C-terminal 12 × HIS-3 × HA-tag with mutated residues M391D, F394D, W402D, W408D of *S. cerevisiae* full length Brn1), TRP1	This work	N/A
Plasmid 2micron pGAL10 YCS4 (*S. cerevisiae* full length Ycs4), URA3	This work	N/A
Plasmid pETMCN-Brn1_384-529_-Ycg1_6-932, Δ499-555_ (N-terminal 6 × HIS-tag-HRV3C-cleavage-site, residues 384-529 of *S. cerevisiae* Brn1 and residues 6-932 with deleted residues 499-555 of untagged *S. cerevisiae* Ycg1)	This work	N/A
Plasmid pETMCN-Brn1_384-529, short kleisin loop_-Ycg1_6-932, Δ499-555_ (N-terminal 6 × HIS-tag-HRV3C-cleavage-site, residues 384-529 with deleted residues 418-444 of *S. cerevisiae* Brn1 and residues 6-932 with deleted residues 499-555 of untagged *S. cerevisiae* Ycg1)	This work	N/A
Plasmid pETMCN-Cnd2_416-544_-Cnd3_1-823, Δ439-473_ (N-terminal 6 × HIS-tag-HRV3C-cleavage-site, residues 416-544 of *S. pombe* Cnd2 and residues 1-823 with deleted residues 439-473 of untagged *S. pombe* Cnd3)	This work	N/A
Plasmid pC1-NCAPH (N-terminal Flag-EGFP-tag of *H. sapiens* NCAPH)	Piazza et al., 2014	N/A
Plasmid pC1-NCAPH2 (N-terminal Flag-EGFP-tag of *H. sapiens* NCAPH2)	Piazza et al., 2014	N/A
Plasmid pC1-NCAPH_BC1/2_ (N-terminal Flag-EGFP-tag with mutations R446D, R448D, R450D, R451D, K452D, K462D, K463D, K464D, K467D, K468D of *H. sapiens* NCAPH)	This work	N/A
Plasmid pC1-NCAPH _**Φ**A_ (N-terminal Flag-EGFP-tag with mutations Y428A, F431A, W439A, W445A of *H. sapiens* NCAPH)	This work	N/A
Plasmid pC1-NCAPH _ΦD_ (N-terminal Flag-EGFP-tag with mutations Y428D, F431D, W439D, W445D of *H. sapiens* NCAPH)	This work	N/A
Plasmid pC1-NCAPH _S432A_ (N-terminal Flag-EGFP-tag with mutations S432A of *H. sapiens* NCAPH)	This work	N/A
Plasmid pC1-NCAPH2_BC1/2_ (N-terminal Flag-EGFP-tag with mutations K329D, K332D, K333D, R335D, K350D, R351D, K352D, R353D, K354D of *H. sapiens* NCAPH2)	This work	N/A

**Software and Algorithms**		

X-ray Detector Software (XDS)	Kabsch, 2010	http://xds.mpimf-heidelberg.mpg.de/
SHELX	Sheldrick, 2008	http://shelx.uni-ac.gwdg.de/SHELX/
Phenix suite	Adams et al., 2010	https://www.phenix-online.org/
CCP4 suite	Winn et al., 2011	http://www.ccp4.ac.uk/
COOT v0.8.2	Emsley et al., 2010	https://www2.mrc-lmb.cam.ac.uk/personal/pemsley/coot/
PyMOL	Schrödinger, LLC	https://www.pymol.org/
ConSurf	Ashkenazy et al., 2016	http://bental.tau.ac.il/new_ConSurfDB/
APBS	Baker et al., 2001	http://www.poissonboltzmann.org/
PISA	Krissinel and Henrick, 2007	http://www.ccp4.ac.uk/MG/ccp4mg_help/pisa.html

### Contact for Reagent and Resource Sharing

Further information and requests for resources and reagents should be directed to and will be fulfilled by the Lead Contact, Christian H. Haering (christian.haering@embl.de).

### Experimental Model and Subject Details

#### Cell lines

Female HeLa Kyoto H2B-mCherry cells (Neumann et al., 2010) were cultivated in DMEM (Life Technologies) containing 10% FBS (Life Technologies), 1% PenStrep (Invitrogen), and 1% glutamine (Invitrogen) at 37°C, 5% CO_2_.

#### Yeast strains

*Saccharomyces cerevisiae* strains are derived of W303. Genotypes of strains C4237, C4239, C4257, C4259, C4261, C4491, C4493, C4516, C4724, C4895 and C5037are listed in the Key Resources Table.

#### Bacterial strains

Proteins for crystallography and biochemistry were expressed in *Escherichia coli* Rosetta (DE3) pLysS cells (Merck, Cat#70954) pre-cultured at 37°C and then shifted to 18°C for induction in 2 × TY or Terrific Broth (TB) medium.

### Method Details

#### Protein expression and purification

Expression of *Ct* Ycg1, *Ct* Ycs4, *Ct* Ycg1–Brn1, or *Ct* Ycs4–Brn1 and the crystallization constructs *Sc* Ycg1–Brn1 or *Sp* Cnd3–Cnd2 was induced for 18 hr from pET-MCN vectors (Romier et al., 2006) in *Escherichia coli* Rosetta (DE3) pLysS (Merck) grown at 18°C in Terrific Broth (TB) medium (for *Sc* Ycg1–Brn1) or 2 × TY medium (for all other constructs). Cells were lysed by sonication at 4°C in lysis buffer (50 mM TRIS–HCl pH 7.5, 500 mM NaCl, 20 mM imidazole, 5 mM β-mercaptoethanol containing cOmplete protease inhibitor cocktail tablets without EDTA (cOm–EDTA, Roche)). The lysate was cleared by centrifugation at 45,000 × g_max_ and loaded onto Ni-Sepharose 6FF (GE Healthcare). After washing with 30-40 column volumes (CV) lysis buffer, proteins were eluted in 5-7 CV elution buffer (lysis buffer plus 300 mM imidazole). The eluate was dialyzed overnight in dialysis buffer (25 mM TRIS–HCl pH 7.5, 300 mM NaCl, 1 mM DTT) at 4°C, diluted with low-salt buffer (25 mM TRIS–HCl pH 7.5, 100 mM NaCl, 1 mM DTT) to a final salt concentration of 150 mM NaCl and loaded onto a 6 mL RESOURCE Q (GE Healthcare) anion exchange column pre-equilibrated with low-salt buffer. After washing with 3-5 CV low-salt buffer, proteins were eluted by increasing NaCl concentrations to 1 M in a linear gradient of 60 mL. Peak fractions were pooled and loaded onto a Superdex 200 26/60 column (GE Healthcare) equilibrated in SEC-buffer (25 mM TRIS–HCl pH 7.5, 500 mM NaCl, 1 mM DTT). Peak fractions were pooled and concentrated by ultrafiltration (Vivaspin 30,000 MWCO, Sartorius).

*Ct* Brn1_336-714_ was expressed as a N-terminal GST fusion construct from a pGEX 6P-1 (Smith and Johnson, 1988) as described above. Cells were lysed at 4°C by sonication in lysis buffer (50 mM TRIS–HCl pH 7.5, 500 mM NaCl, 2 mM DTT containing cOm–EDTA). The lysate was cleared by centrifugation at 45,000 × g _max_ and loaded onto Glutathione Sepharose 4B beads (GE Healthcare). The GST-fusion protein was eluted from the beads with lysis buffer containing 10 mM L-glutathione. The eluate was dialyzed and purified over RESOURCE Q as described above. Peak fractions were pooled, concentrated by ultrafiltration (Vivaspin 10,000 MWCO) and loaded on a Superdex 200 10/30 column (GE Healthcare) equilibrated in SEC buffer (25 mM TRIS–HCl pH 7.5, 500 mM NaCl, 1 mM DTT). Peak fractions were pooled and concentrated by ultrafiltration (Vivaspin 10,000 MWCO).

Trimeric *Ct* Ycg1–Ycs4–Brn1 complexes were prepared by mixing purified *Ct* Ycs4 at 1.1-fold molar excess with purified *Ct* Ycg1–Brn1_225-634_ subcomplexes and 30 min incubation on ice. An equimolar complex was separated via 6 mL RESOURCE Q as described above. Peak fractions were pooled and concentrated by ultrafiltration (Vivaspin 30,000 MWCO).

Condensin holocomplexes (*Sc* Smc2, Smc4-StrepII_3_, Brn1-His_12_-HA_3_, Ycg1, Ycs4; *Sc* Smc2, Smc4-StrepII_3_, Brn1-His_12_-HA_3_, Ycs4; *Sc* Smc2, Smc4-StrepII_3_, Brn1_BC1/2_-His_12_-HA_3_, Ycg1, Ycs4; *Sc* Smc2, Smc4-StrepII_3_, Brn1_ΦD_-His_12_-HA_3_, Ycg1, Ycs4 and *Sc* Smc2_Q147L_, Smc4_Q302L_-StrepII_3_, Brn1-His_12_-HA_3_, Ycg1, Ycs4) were expressed in *Sc* (strains C4491, C4493, C4516, C5037 and C4724) and purified as described previously (St-Pierre et al., 2009). Expression was induced with 2% (w/v) galactose in synthetic complete (SC) –TRP –URA medium for 14-18 hr at 30°C. Cells were harvested and lysed by cryogenic grinding (SPEX Sample Prep Freezer/Mill 6970). Lysates were thawed in lysis buffer (50 mM TRIS–HCl pH 7.5, 200 mM NaCl, 20 mM imidazole, 5 mM β-mercaptoethanol, 1 mM MgCl_2_, 5% (v/v) glycerol) containing cOm–EDTA and cleared by centrifugation at 45,000xg_max_. The cleared lysate was loaded on a 5 mL HisTrap HP column (GE Healthcare), washed with lysis buffer containing 500 mM NaCl and lysis buffer containing 40 mM imidazole and eluted in elution buffer (50 mM TRIS–HCl pH 7.5, 200 mM NaCl, 200 mM imidazole, 5 mM β-mercaptoethanol, 1 mM MgCl_2_, 5% (v/v) glycerol). The eluate was supplemented with 1.5 mM EDTA, 0.01% (v/v) Tween20, 0.1 mM PMSF and incubated with 1 mL Strep-Tactin Superflow high capacity beads (IBA) at 4°C for 16 hr. Beads were washed in SB-wash buffer (50 mM TRIS–HCl pH 7.5, 500 mM NaCl, 1 mM DTT, 1 mM MgCl_2_, 5% (v/v) glycerol, 0.01% (v/v) Tween20). For complexes used for ATPase assays, beads were washed additionally with SB-ATP-wash buffer (50 mM TRIS–HCl pH 7.5, 200 mM NaCl, 50 mM KCl, 1 mM ATP, 1 mM DTT, 10 mM MgCl_2_, 5% (v/v) glycerol, 0.01% (v/v) Tween20). The protein was eluted in SB-elution buffer (50 mM TRIS–HCl pH 7.5, 200 mM NaCl, 1 mM DTT, 1 mM MgCl_2_, 5% (v/v) glycerol, 10 mM d-Desthiobiotin). Eluate fractions were concentrated by ultrafiltration (Vivaspin 30,000 MWCO) and loaded on a Superose 6 10/30 GL column (GE Healthcare) equilibrated in Sup6 buffer (50 mM TRIS–HCl pH 7.5, 200 mM NaCl, 1 mM DTT, 1 mM MgCl_2_, 5% (v/v) glycerol). Peak fractions were pooled and concentrated by ultrafiltration (Vivaspin 30,000 MWCO).

Selenomethionine-labeled *Sp* Cnd2-Cnd3 was expressed applying methionine pathway inhibition (Doublié, 1997) and purified as described above.

#### Crystallization and data collection

Crystals of selenomethionine-labeled and native *Sp* Cnd3–Cnd2 (Table S1) were grown at 7°C by hanging-drop vapor diffusion. Volumes of 1 μL protein (7-12 mg/mL in 10 mM TRIS–HCl 7.5, 100 mM NaCl, 1 mM DTT) was mixed with 1 μL crystallization solution 1 (18% (w/v) Sokalan CP 42 (Molecular Dimensions), 0.1 M BIS–TRIS pH 5.8, 0.1 M Li acetate) for labeled or with 1 μL crystallization solution 2 (3%–4% (w/v) PEG 4,000, 0.1 M Na citrate pH 5.2-5.4, 0.2 M Na acetate) for native protein. For the selenomethionine-labeled crystals, drops were pre-equilibrated for one day at 7°C before streak seeding with previously grown and crushed crystal clusters. Crystals were harvested after 7-9 days and cryo-protected by addition of crystallization solution containing 25% (v/v) glycerol and 25% (w/v) Sokalan CP 42 (labeled) or 30% (v/v) PEG 400 (native) before flash freezing in liquid nitrogen. Single-wavelength anomalous dispersion data were collected at a wavelength of 0.979 Å (peak) and native data at 0.977 Å at beamline ID29, European Synchrotron Radiation Facility (ESRF, Grenoble, France) (de Sanctis et al., 2012). Data were processed with XDS (Kabsch, 2010) and POINTLESS (Evans, 2006, Evans, 2011) and scaled and merged with AIMLESS of the CCP4 suite (Evans and Murshudov, 2013, Winn et al., 2011).

*Sc* Ycg1–Brn1 crystals (Table S1) were grown by hanging-drop vapor diffusion after mixing 1 μL protein (8 mg/mL in 10 mM TRIS–HCl pH 7.5, 300 mM NaCl, 1 mM DTT) and 1 μL crystallization solution (3% (w/v) PEG 4,000, 0.1 M Na citrate pH 5.5, 0.2 M Na acetate) at 20°C before streak seeding with previously grown and crushed crystal clusters. Crystals were harvested 15 days after setup and cryo-protected by addition of 35% (v/v) PEG 400 before flash freezing in liquid nitrogen. The dataset was collected at a wavelength of 1.000 Å at beamline ID29, ESRF (de Sanctis et al., 2012). Data were processed as described above with the exception of using SCALA (Evans, 2006) instead of AIMLESS.

*Sc* Ycg1–Brn1–DNA (I, II and short kleisin loop, Table S1) crystals were grown by sitting drop vapor diffusion after mixing 100 nL sample and 100 nL crystallization solution in an MRC 2-well plate (Hampton Research). dsDNA was prepared by annealing a palindromic 18 bp HPLC-purified DNA oligo (IDT, GATGTGTAGCTACACATC, modified from Seifert et al., 2016) in annealing buffer (10 mM TRIS–HCl pH 7.5, 130 mM NaCl, 1 mM DTT) in a temperature gradient of 0.1°C/s from 95°C to 4°C. The crystallization sample contained 1.2-fold molar excess of DNA over protein. For *Sc* Ycg1–Brn1-DNA (I), protein (5 mg/mL in 10 mM TRIS–HCl pH 7.5, 250 mM NaCl, 1 mM DTT) and DNA were mixed with crystallization buffer (4% (w/v) PEG 4,000, 0.05 M Na citrate pH 6.6, 5 mM MgSO_4_) and crystals were harvested after 15 days at 7°C. For *Sc* Ycg1–Brn1–DNA (II), protein (5 mg/mL in 10 mM TRIS–HCl pH 7.5, 250 mM NaCl, 1 mM DTT) and DNA were mixed with crystallization buffer (5% (w/v) PEG 4,000, 0.05 M Na cacodylate pH 6.8, 10 mM BaCl_2_) and crystals were harvested after 9 days at 7°C. For *Sc* Ycg1–Brn1_short kleisin loop_–DNA, protein (6 mg/mL in 10 mM TRIS–HCl pH 7.5, 250 mM NaCl, 1 mM DTT) and DNA were mixed with crystallization buffer (4% (w/v) PEG 4,000, 0.05 M Na citrate pH 5.6, 5 mM MgSO_4_) and crystals were harvested after 12 days at 7°C. Crystals were flash frozen in liquid nitrogen after addition of 2 μL crystallization buffer containing 37.5% (v/v) PEG 200. Datasets of *Sc* Ycg1–Brn1–DNA (I and II) were collected at a wavelength of 0.873 Å at beamline ID23-2, ESRF (Flot et al., 2010). The dataset of *Sc* Ycg1–Brn1_short kleisin loop_–DNA was collected at a wavelength of 0.979 Å at beamline ID29, ESRF (de Sanctis et al., 2012). All datasets were processed as described above using SCALA (Evans, 2006).

#### Structure determination and refinement

Single anomalous dispersion data for *Sp* Cnd3–Cnd2, merged from 4 independent datasets, were used to locate 26 selenium sites with SHELX (Sheldrick, 2008) followed by site refinement, phasing, and density modification. An initial model was built using Phenix AutoBuild and manual adjustment in Coot (Adams et al., 2010, Emsley et al., 2010, Terwilliger et al., 2008). The higher resolution, native Cnd3–Cnd2 dataset was solved by molecular replacement searching with the initial Cnd3–Cnd2 SeMet model using Phenix Phaser-MR (McCoy, 2007). The model was built by iterative rounds of manual adjustments with Coot and of restrained refinements with phenix.refine (Afonine et al., 2012, Emsley et al., 2010).

The *Sc* Ycg1–Brn1 structure was solved by molecular replacement with an adapted *Sp* Cnd3–Cnd2 as search model using Phenix Phaser-MR (McCoy, 2007). An initial model was built using Phenix AutoBuild and manual adjustments with Coot (Emsley et al., 2010, Terwilliger et al., 2008). The structure was further improved in iterative rounds of manual correction with *Coot* and restrained refinements with phenix.refine (Afonine et al., 2012, Emsley et al., 2010).

The *Sc* Ycg1–Brn1–DNA (I) structure was solved by molecular replacement searching with the *Sc* Ycg1–Brn1 model using Phenix Phaser-MR (McCoy, 2007). The model was built in iterative rounds using density modified maps of Phenix AutoBuild and manual adjustments in Coot (Emsley et al., 2010, Terwilliger et al., 2008). The structure was finalized in iterative rounds of manual adjustments with Coot and restrained refinements with phenix.refine (Afonine et al., 2012, Emsley et al., 2010). *Sc* Ycg1–Brn1–DNA (II) and *Sc* Ycg1–Brn1_short kleisin loop_–DNA models were solved by molecular replacement searching with *Sc* Ycg1–Brn1–DNA (I) model using Phenix Phaser-MR (McCoy, 2007). The models were built and refined as described for *Sc* Ycg1–Brn1–DNA (I).

All structures were refined with hydrogens (‘riding’ model) and validated using MolProbity (Chen et al., 2010) (Table S1).

Structures were visualized with PyMOL (Schrödinger, LLC). Surface conservation graphics were created using the ConSurf server (Ashkenazy et al., 2016) using a specified multi-sequence alignment (see below). The electrostatic surface potential graph was created with APBS (Baker et al., 2001). Structural alignments with cohesin-associated HEAT-repeat subunits were performed with the SUPERPOSE program of CCP4 (Krissinel and Henrick, 2007).

#### Electrophoretic mobility shift assay

The 6-FAM labeled 35-bp dsDNA was prepared by annealing two complementary HPLC-purified DNA oligos (IDT, 5′-6-FAM-CCTATAGTGAGTCGTTCGATATTACAATTCACTGG-3′; 5′-CCAGTGAATTGTAATATCGAACGACTCACTATAGG-3′) in annealing buffer (10 mM HEPES–KOH pH 7.5, 125 mM NaCl, 5 mM MgCl_2_) at a concentration of 20 μM in a temperature gradient of 0.1°C/s from 95°C to 4°C. The EMSA reaction with 35-bp dsDNA or 2.1-kb circular, supercoiled plasmid was prepared with a constant DNA concentration of 0.2 μM or 10 nM, respectively, and various concentrations of purified protein in binding buffer (10 mM HEPES–KOH pH 7.5, 125 mM NaCl, 5 mM MgCl_2_, 1 mM DTT). After 30 min incubation and addition of 3.5% (v/v) glycerol, free DNA and DNA–protein complexes were resolved by electrophoresis for 6-14 hr at 6-2 V/cm, respectively, on 1.8% (w/v) for short dsDNA or 0.8% (w/v) for 2.1-kb circular plasmid DNA TAE-agarose gels at 4°C. Gels with 6-FAM labeled short dsDNA were detected directly, gels with 2.1-kb plasmid DNA were stained with ethidium bromide prior to detection on a Typhoon FLA 9,500 scanner (GE Healthcare) with excitation at 473 nm with LPB (510LP) or at 532 nm with LPG (575LP) filter setting, respectively.

#### Cysteine-crosslinking of Brn1

Reactions with 2.1-kb supercoiled circular or linear DNA substrates were prepared at 14.4 nM (20 ng/μL) concentrations and 10 μM wild-type or cysteine-containing *Ct* Ycg1–Brn1_515-634_ or 2.5 μM wild-type or cysteine-containing *Ct* Ycs4–Ycg1–Brn1_225-634_. DNA and protein samples were re-buffered into crosslinking buffer (1 × PBS, 5 mM MgCl_2_) prior to mixing and incubated for 30 min on ice. 10% (v/v) DMSO, 30 μM dibromobimane (bBBr in DMSO, Sigma-Aldrich), 30 μM disthiobismaleimidoethane (DTME in DMSO, ThermoFisher Scientific) or 30 μM bismaleimidoethane (BMOE in DMSO, ThermoFisher Scientific) were added to the reaction followed by incubation for 10 min on ice. The crosslinking reaction was stopped by addition of 5 mM DTT for 5 min on ice for bBBr and BMOE. DTT was either not added or added to 55.5 mM for the DTME crosslinking reaction. Brn1 cleavage at the HRV-3C site was induced by addition of 1 μg HRV-3C protease and incubation for 25 min at 7°C. Proteins were denatured by addition of SDS to 1% (w/v) and incubation for 10 min at 65°C. Samples were resolved by electrophoresis for 14 hr at 2 V/cm on 0.8% (w/v) TAE agarose gels at 4°C, followed by detection of DNA by ethidium bromide staining.

Crosslinking experiments of cysteine containing *Ct* Ycg1-Brn1_515-634_ with and without HRV-3C site followed by SDS-PAGE and Coomassie staining were performed as described above for the upshift experiments of circular DNA with the exception of incubating 10 μM protein with 20 μM annealed, palindromic 22-bp dsDNA (IDT, 5′-GATTCGTGTAGCTACACGAATC-3′, Seifert et al., 2016) in all reactions. Crosslinking experiments of cysteine containing *Ct* Ycs4–Ycg1–Brn1_225-634_ with BMOE prior EMSA and incubation with 2.1-kb supercoiled-circular DNA was done as described above without 22-bp dsDNA incubation before crosslinking.

#### ATP hydrolysis assays

Reactions (10 μL) were set up with 0.5 μM condensin holocomplex, with or without 24 nM relaxed circular 6.4-kb plasmid DNA in ATPase buffer (40 mM TRIS–HCl pH 7.5, 125 mM NaCl, 10% (v/v) glycerol, 5 mM MgCl_2_, 5 mM ATP, 1 mM DTT and 33 nM [α^32^P]-ATP; Hartmann Analytic). Relaxed circular DNA was prepared by treating the negatively supercoiled plasmid DNA with *E. coli* topoisomerase I (NEB). After treatment with topo I, relaxed DNA was purified by phenol-chloroform extraction and ethanol precipitation. ATP hydrolysis reactions were incubated at RT (∼25°C) and were initiated by addition of ATP. A volume of 0.8 μL of the reaction mix was spotted onto PEI cellulose F TLC plates (Merck) every 3 min for a total duration of 15 min. The reaction products were resolved on TLC plates using 0.5 M LiCl and 1 M formic acid solution and detected by exposing the TLC plates to a phosphorimager screen and analysis on a Typhoon FLA 9,500 scanner (GE Healthcare). ATP hydrolysis rates were calculated from the ADP/ATP ratios from time points in the linear range of the reaction.

#### Isothermal titration calorimetry

The 25-bp dsDNA substrate was prepared by annealing two complementary HPLC-purified DNA oligonucleotides (IDT, 5′-CCTATAGTGAGTCACAATTCACTGG-3′; 5′- CCAGTGAATTGTGACTCACTATAGG-3′) in annealing buffer (10 mM HEPES–KOH pH 7.5, 125 mM NaCl, 5 mM MgCl_2_) at a concentration of 20 μM using a temperature gradient of 0.1°C/s from 95°C to 4°C. The DNA was then re-buffered into ITC buffer (25 mM TRIS–HCl pH 7.5, 200 mM NaCl, 2 mM MgCl_2_, 1 mM BME) by multiple rounds of concentration and dilution using ultrafiltration (Vivaspin 3,000 MWCO). *Ct* Ycg1–Brn1 was dialyzed to ITC buffer. *Ct* Brn1-Ycg1 was injected with a concentration of 250 μM into 10 μM 25-bp dsDNA at 25°C.

ITC measurements were performed on a MicroCal iTC200 microcalorimeter (GE Healthcare). The ITC data were corrected for the dilution heat and fitted with the MicroCal Origin software package applying one set of binding sites model. Standard deviation values of the fit were calculated from the original data points.

#### Condensin immunoprecipitation and western blotting

Immunoprecipitation of endogenous condensin complexes from yeast was performed as described previously (Piazza et al., 2014). Yeast strains C4237, C4239 and C4261 were grown at 30°C in 2 l YPAD to OD_600_ = 1, harvested by centrifugation and lysed by cryogenic grinding (SPEX Sample Prep Freezer/Mill 6970) in lysis buffer (50 mM TRIS–HCl pH 8.0, 100 mM NaCl, 2.5 mM MgCl_2_, 0.25% (v/v) Triton X-100, 1 mM DTT, 1 mM PMSF) containing 2 × cOm–EDTA. The lysate was cleared by centrifugation at 20,400 × g_max_ and incubated with 100 μL protein A coupled Dynabeads (ThermoFisher Scientific) that were previously bound to anti-PK (V5) tag (Abd Serotec, MCA1360) antibody for 2 hr at 4°C. Beads were washed with IP buffer (50 mM TRIS–HCl pH 8.0, 100 mM NaCl, 1 mM DTT, 5 mM EDTA, 0.25% (v/v) Triton X-100) and eluted in 20 μL 2 × SDS loading buffer (100 mM TRIS–HCl pH 6.8, 4% (w/v) SDS, 20% (v/v) glycerol, 0.2% (w/v) bromophenol blue, 0.2 M DTT) by boiling at 95°C for 5 min prior to SDS-PAGE and Coomassie staining or western blotting with antibodies against the PK (V5) tag (Abd Serotec, MCA1360), *Sc* Ycg1 (Piazza et al., 2014) or α-tubulin (TAT1) (Woods et al., 1989).

#### ChIP-qPCR

ChIP-qPCR experiments were performed as described previously (Cuylen et al., 2011). Yeast strains C4237, C4239 and C4261 were grown in 42 mL YPAD at 30°C to OD_600_ = 0.6 and fixed with 4.7 mL fixation buffer (9.5 mM TRIS–HCl pH 8.0, 19 mM NaCl, 0.095 mM EGTA, 3% (v/v) formaldehyde, 0.19 mM EDTA) for 30 min at 16°C. Fixation was stopped by addition of glycine to 125 mM (final concentration), followed by washing steps in PBS and PIPES buffer (100 mM PIPES–KOH pH 8.3). Cells were lysed by spheroplasting with 0.5 mg/mL zymolase T-100 (AMS Biotechnology) in HEMS buffer (100 mM HEPES–KOH pH 7.5, 1 mM EGTA, 1 mM MgSO_4_, 1.2 M Sorbitol, 1 mM PMSF containing cOm–EDTA), followed by resuspension of cells in 1.5 mL lysis buffer (50 mM HEPES–KOH pH 7.5, 140 mM NaCl, 1 mM EDTA, 1% (v/v) Triton X-100, 0.1% (w/v) sodium deoxycholate, 1 mM PMSF containing cOm–EDTA). Chromatin was sheared by sonication to a length of ∼500 bp using a Bioruptor UCD-200 (Diagenode) for 9 min, 30 s on, 60 s off settings (‘high level’).

Lysate was cleared by centrifugation at 16,800 × g_max_ and pre-cleared with 50 μL protein A dynabeads (ThermoFisher Scientific) for 90 min at 4°C. 10% of the cleared lysate was used to check sonication, 12% was kept on ice as input sample. 2 μg anti-PK (V5) tag antibody (Abd Serotec MCA1360) was added to the remaining lysate and samples were incubated at 4°C for 16 hr before addition of 100 μL protein A dynabeads (ThermoFisher Scientific) for another 4 hr at 4°C. Beads were washed with lysis buffer, wash buffer (10 mM TRIS–HCl pH 8.0, 0.25 M LiCl, 0.5% (w/v) sodium deoxycholate, 1 mM EDTA, 1 mM PMSF containing cOm–EDTA) and TE buffer (10 mM TRIS–HCl pH 8.0, 1 mM EDTA containing cOm–EDTA). Samples were eluted in 320 μL TES buffer (50 mM TRIS–HCl pH 8.0, 10 mM EDTA, 1% (w/v) SDS) at 65°C for 8 hr. After addition of 30 μg RNaseA (Roche) for 90 min at 37°C and 200 μg Proteinase K (Roche) for 90 min at 65°C, DNA was purified via a spin column (QIAGEN) and eluted in 50 μL EB buffer.

qPCR reactions were set up for 5 μL of 1:5 and 1:25 dilutions for immunoprecipitated samples and 1:5, 1:50, 1:500 and 1:5,000 dilutions for input samples with SYBR green PCR Master mix (Applied Biosystems) and 5 μM qPCR primers (5′-TGGTGTGGAAGTCCTAATATCG-3′ and 5′-TGCATGATCAAAAGGCTCAA-3′ for *CEN4* and 5′- TTTCTGCCTTTTTCGGTGAC-3′ and 5′- TGGCATGGATTTCCCTTTAG-3′ for *rDNA*) on an Applied Biosystems 7,500 Fast Real-Time PCR System. Data were calculated from two independent experiments with two qPCR runs each.

#### Microscopy of human condensin complexes

Female HeLa Kyoto H2B-mCherry cells (Neumann et al., 2010) were transiently transfected with pC1 Flag-EGFP-NCAPH or Flag-EGFP-NCAPH2 as described previously (Piazza et al., 2014), with the following modifications: Transfections were performed in Lab-Tek dishes (ThermoFisher Scientific) by addition of transfection mix (400 ng plasmid DNA and 1.5 μL FuGene (Promega) in 50 μL Opti-MEM medium (Life Technologies)) at 50% confluency (1 × 10^5^ cells). After 12-16 hr, the medium was replaced with high-glucose DMEM (Life Technologies) containing 10% FBS (Life Technologies), 1% PenStrep (Invitrogen), and 1% glutamine (Invitrogen). 5-12 hr before imaging, fresh medium supplemented with 250 ng/mL nocodazole was added. Cells were washed once and imaged 36 hr after transfection in medium without phenol red containing 250 ng/mL nocodazole. Image acquisition was performed on a Zeiss LSM 780 microscope in 16-bit mode and four lines averaging with a Plan-Apochromat 63 × /1.40 oil DIC M27 objective at 37°C and 5% CO_2_. Excitation and emission wavelengths were 488 nm and 520-560 nm or 561 nm and 580-650 nm for EGFP or mCherry, respectively. The cell line was authenticated and tested for mycoplasma contamination.

Images were analyzed with Fiji (Schindelin et al., 2012). First, background was subtracted using the rolling ball algorithm. Chromatin regions were segmented based on the mCherry fluorescence signal and the whole cell was segmented based on the bright field image. Cytoplasmic regions were selected after subtracting the areas of chromatin from the whole cell regions. Mean fluorescence intensities of EGFP images were measured for chromatin and cytoplasmic regions. Data were calculated from two independent experiments.

#### Multiple-sequence alignments

Brn1 and Ycg1 sequences were aligned from 35 divergent species (10 plants, 10 fungi, 10 animals, 5 protists; Table S2) with MAFFT (Katoh et al., 2002) using the Smith-Waterman local algorithm (L-INS-i) with default settings and gaps were manually optimized. Conserved residues were highlighted using the ClustalW color code.

### Quantification and Statistical Analysis

Statistical details of experiments can be found in the figure legends or Method Details section.

### Data and Software Availability

Crystal structures have been deposited in the Protein Data Bank (https://www.rcsb.org/pdb) under ID codes PBD: 5OQR (Sp Cnd3–Cnd2), PBD: 5OQQ (Sc Ycg1–Brn1), PBD: 5OQP (Sc Ycg1–Brn1-DNA (I)), PBD: 5OQO (Sc Ycg1–Brn1–DNA (II)), and PBD: 5OQN (Sc Ycg1–Brn1short kleisin loop–DNA).

## Author Contributions

M.K. purified proteins and conducted biochemical assays. M.K. and M.H. crystallized the proteins, collected data, and solved the structures. F.M. carried out imaging experiments with human cells. M.K. and J.M. conducted functional assays in yeast. J.M. performed ChIP-qPCR experiments. S.B. provided purified condensin holocomplexes and performed ATPase assays. V.R. performed ITC experiments. M.K., M.H., and C.H.H. designed the study. C.H.H. supervised the work. M.K, M.H., and C.H.H. wrote the manuscript with input from all authors.

## References

[bib1] Adams P.D., Afonine P.V., Bunkóczi G., Chen V.B., Davis I.W., Echols N., Headd J.J., Hung L.W., Kapral G.J., Grosse-Kunstleve R.W. (2010). PHENIX: a comprehensive Python-based system for macromolecular structure solution. Acta Crystallogr. D Biol. Crystallogr..

[bib2] Afonine P.V., Grosse-Kunstleve R.W., Echols N., Headd J.J., Moriarty N.W., Mustyakimov M., Terwilliger T.C., Urzhumtsev A., Zwart P.H., Adams P.D. (2012). Towards automated crystallographic structure refinement with phenix.refine. Acta Crystallogr. D Biol. Crystallogr..

[bib3] Alipour E., Marko J.F. (2012). Self-organization of domain structures by DNA-loop-extruding enzymes. Nucleic Acids Res..

[bib4] Anderson D.E., Losada A., Erickson H.P., Hirano T. (2002). Condensin and cohesin display different arm conformations with characteristic hinge angles. J. Cell Biol..

[bib5] Andrade M.A., Bork P. (1995). HEAT repeats in the Huntington’s disease protein. Nat. Genet..

[bib6] Ashkenazy H., Abadi S., Martz E., Chay O., Mayrose I., Pupko T., Ben-Tal N. (2016). ConSurf 2016: an improved methodology to estimate and visualize evolutionary conservation in macromolecules. Nucleic Acids Res..

[bib7] Baker N.A., Sept D., Joseph S., Holst M.J., McCammon J.A. (2001). Electrostatics of nanosystems: application to microtubules and the ribosome. Proc. Natl. Acad. Sci. USA.

[bib8] Chen V.B., Arendall W.B., Headd J.J., Keedy D.A., Immormino R.M., Kapral G.J., Murray L.W., Richardson J.S., Richardson D.C. (2010). MolProbity: all-atom structure validation for macromolecular crystallography. Acta Crystallogr. D Biol. Crystallogr..

[bib9] Ciosk R., Shirayama M., Shevchenko A., Tanaka T., Toth A., Shevchenko A., Nasmyth K. (2000). Cohesin’s binding to chromosomes depends on a separate complex consisting of Scc2 and Scc4 proteins. Mol. Cell.

[bib10] Cook A.G., Fukuhara N., Jinek M., Conti E. (2009). Structures of the tRNA export factor in the nuclear and cytosolic states. Nature.

[bib11] Cuylen S., Metz J., Haering C.H. (2011). Condensin structures chromosomal DNA through topological links. Nat. Struct. Mol. Biol..

[bib12] de Sanctis D., Beteva A., Caserotto H., Dobias F., Gabadinho J., Giraud T., Gobbo A., Guijarro M., Lentini M., Lavault B. (2012). ID29: a high-intensity highly automated ESRF beamline for macromolecular crystallography experiments exploiting anomalous scattering. J. Synchrotron Radiat..

[bib13] Dekker J., Mirny L. (2016). The 3D Genome as Moderator of Chromosomal Communication. Cell.

[bib14] Dephoure N., Zhou C., Villén J., Beausoleil S.A., Bakalarski C.E., Elledge S.J., Gygi S.P. (2008). A quantitative atlas of mitotic phosphorylation. Proc. Natl. Acad. Sci. USA.

[bib15] Doublié S. (1997). Preparation of selenomethionyl proteins for phase determination. Methods Enzymol..

[bib16] Earnshaw W.C., Laemmli U.K. (1983). Architecture of metaphase chromosomes and chromosome scaffolds. J. Cell Biol..

[bib17] Eeftens J.M., Bisht S., Kerssemakers J., Haering C., Dekker C. (2017). Real-time detection of condensin-driven DNA compaction reveals a multistep binding mechanism. bioRxiv.

[bib18] Emsley P., Lohkamp B., Scott W.G., Cowtan K. (2010). Features and development of Coot. Acta Crystallogr. D Biol. Crystallogr..

[bib19] Eng T., Guacci V., Koshland D. (2015). Interallelic complementation provides functional evidence for cohesin-cohesin interactions on DNA. Mol. Biol. Cell.

[bib20] Evans P. (2006). Scaling and assessment of data quality. Acta Crystallogr. D Biol. Crystallogr..

[bib21] Evans P.R. (2011). An introduction to data reduction: space-group determination, scaling and intensity statistics. Acta Crystallogr. D Biol. Crystallogr..

[bib22] Evans P.R., Murshudov G.N. (2013). How good are my data and what is the resolution?. Acta Crystallogr. D Biol. Crystallogr..

[bib23] Flot D., Mairs T., Giraud T., Guijarro M., Lesourd M., Rey V., van Brussel D., Morawe C., Borel C., Hignette O. (2010). The ID23-2 structural biology microfocus beamline at the ESRF. J. Synchrotron Radiat..

[bib24] Goloborodko A., Imakaev M.V., Marko J.F., Mirny L. (2016). Compaction and segregation of sister chromatids via active loop extrusion. eLife.

[bib25] Griese J.J., Witte G., Hopfner K.-P. (2010). Structure and DNA binding activity of the mouse condensin hinge domain highlight common and diverse features of SMC proteins. Nucleic Acids Res..

[bib26] Hara K., Zheng G., Qu Q., Liu H., Ouyang Z., Chen Z., Tomchick D.R., Yu H. (2014). Structure of cohesin subcomplex pinpoints direct shugoshin-Wapl antagonism in centromeric cohesion. Nat. Struct. Mol. Biol..

[bib27] Hirano T. (2016). Condensin-Based Chromosome Organization from Bacteria to Vertebrates. Cell.

[bib28] Hirano T., Kobayashi R., Hirano M. (1997). Condensins, chromosome condensation protein complexes containing XCAP-C, XCAP-E and a Xenopus homolog of the Drosophila Barren protein. Cell.

[bib29] Houlard M., Godwin J., Metson J., Lee J., Hirano T., Nasmyth K. (2015). Condensin confers the longitudinal rigidity of chromosomes. Nat. Cell Biol..

[bib30] Ivanov D., Nasmyth K. (2005). A topological interaction between cohesin rings and a circular minichromosome. Cell.

[bib31] Kabsch W. (2010). XDS. Acta Crystallogr. D Biol. Crystallogr..

[bib32] Katoh K., Misawa K., Kuma K., Miyata T. (2002). MAFFT: a novel method for rapid multiple sequence alignment based on fast Fourier transform. Nucleic Acids Res..

[bib33] Kikuchi S., Borek D.M., Otwinowski Z., Tomchick D.R., Yu H. (2016). Crystal structure of the cohesin loader Scc2 and insight into cohesinopathy. Proc. Natl. Acad. Sci. USA.

[bib34] Kimura K., Hirano T. (2000). Dual roles of the 11S regulatory subcomplex in condensin functions. Proc. Natl. Acad. Sci. USA.

[bib35] Kinoshita K., Kobayashi T.J., Hirano T. (2015). Balancing acts of two HEAT subunits of condensin I support dynamic assembly of chromosome axes. Dev. Cell.

[bib36] Krissinel E., Henrick K. (2007). Inference of macromolecular assemblies from crystalline state. J. Mol. Biol..

[bib37] Kschonsak M., Haering C.H. (2015). Shaping mitotic chromosomes: From classical concepts to molecular mechanisms. BioEssays.

[bib38] Lee B.-G., Roig M.B., Jansma M., Petela N., Metson J., Nasmyth K., Löwe J. (2016). Crystal Structure of the Cohesin Gatekeeper Pds5 and in Complex with Kleisin Scc1. Cell Rep..

[bib39] McCoy A.J. (2007). Solving structures of protein complexes by molecular replacement with Phaser. Acta Crystallogr. D Biol. Crystallogr..

[bib40] Nasmyth K. (2001). Disseminating the genome: joining, resolving, and separating sister chromatids during mitosis and meiosis. Annu. Rev. Genet..

[bib41] Naumova N., Imakaev M., Fudenberg G., Zhan Y., Lajoie B.R., Mirny L.A., Dekker J. (2013). Organization of the mitotic chromosome. Science.

[bib42] Neumann B., Walter T., Hériché J.-K., Bulkescher J., Erfle H., Conrad C., Rogers P., Poser I., Held M., Liebel U. (2010). Phenotypic profiling of the human genome by time-lapse microscopy reveals cell division genes. Nature.

[bib43] Neuwald A.F., Hirano T. (2000). HEAT repeats associated with condensins, cohesins, and other complexes involved in chromosome-related functions. Genome Res..

[bib44] Okada C., Yamashita E., Lee S.J., Shibata S., Katahira J., Nakagawa A., Yoneda Y., Tsukihara T. (2009). A high-resolution structure of the pre-microRNA nuclear export machinery. Science.

[bib45] Olsen J.V., Vermeulen M., Santamaria A., Kumar C., Miller M.L., Jensen L.J., Gnad F., Cox J., Jensen T.S., Nigg E.A. (2010). Quantitative phosphoproteomics reveals widespread full phosphorylation site occupancy during mitosis. Sci. Signal..

[bib46] Onn I., Aono N., Hirano M., Hirano T. (2007). Reconstitution and subunit geometry of human condensin complexes. EMBO J..

[bib47] Petrova B., Dehler S., Kruitwagen T., Hériché J.-K., Miura K., Haering C.H. (2013). Quantitative analysis of chromosome condensation in fission yeast. Mol. Cell. Biol..

[bib48] Piazza I., Rutkowska A., Ori A., Walczak M., Metz J., Pelechano V., Beck M., Haering C.H. (2014). Association of condensin with chromosomes depends on DNA binding by its HEAT-repeat subunits. Nat. Struct. Mol. Biol..

[bib49] Rana V., Bosco G. (2017). Condensin Regulation of Genome Architecture. J. Cell. Physiol..

[bib50] Robellet X., Vanoosthuyse V., Bernard P. (2017). The loading of condensin in the context of chromatin. Curr. Genet..

[bib51] Rohs R., Jin X., West S.M., Joshi R., Honig B., Mann R.S. (2010). Origins of specificity in protein-DNA recognition. Annu. Rev. Biochem..

[bib52] Romier C., Ben Jelloul M., Albeck S., Buchwald G., Busso D., Celie P.H.N., Christodoulou E., De Marco V., van Gerwen S., Knipscheer P. (2006). Co-expression of protein complexes in prokaryotic and eukaryotic hosts: experimental procedures, database tracking and case studies. Acta Crystallogr. D Biol. Crystallogr..

[bib53] Rubinson E.H., Gowda A.S.P., Spratt T.E., Gold B., Eichman B.F. (2010). An unprecedented nucleic acid capture mechanism for excision of DNA damage. Nature.

[bib54] Schindelin J., Arganda-Carreras I., Frise E., Kaynig V., Longair M., Pietzsch T., Preibisch S., Rueden C., Saalfeld S., Schmid B. (2012). Fiji: an open-source platform for biological-image analysis. Nat. Methods.

[bib55] Seifert F.U., Lammens K., Stoehr G., Kessler B., Hopfner K.-P. (2016). Structural mechanism of ATP-dependent DNA binding and DNA end bridging by eukaryotic Rad50. EMBO J..

[bib56] Sheldrick G.M. (2008). A short history of SHELX. Acta Crystallogr A..

[bib57] Sironi L., Mapelli M., Knapp S., De Antoni A., Jeang K.-T., Musacchio A. (2002). Crystal structure of the tetrameric Mad1-Mad2 core complex: implications of a ‘safety belt’ binding mechanism for the spindle checkpoint. EMBO J..

[bib58] Smith D.B., Johnson K.S. (1988). Single-step purification of polypeptides expressed in Escherichia coli as fusions with glutathione S-transferase. Gene.

[bib59] St-Pierre J., Douziech M., Bazile F., Pascariu M., Bonneil E., Sauvé V., Ratsima H., D’Amours D. (2009). Polo kinase regulates mitotic chromosome condensation by hyperactivation of condensin DNA supercoiling activity. Mol. Cell.

[bib60] Stray J.E., Lindsley J.E. (2003). Biochemical analysis of the yeast condensin Smc2/4 complex: an ATPase that promotes knotting of circular DNA. J. Biol. Chem..

[bib61] Strick, T.R., Kawaguchi, T., and Hirano, T. (2004). Real-time detection of single-molecule DNA compaction by condensin I. *14*, 874–880. 10.1016/j.cub.2004.04.038 15186743

[bib62] Terekawa T., Bisht S., Eeftens J., Dekker C., Haering C., Greene E. (2017). The Condensin Complex Is A Mechanochemical Motor That Translocates Along DNA. Science.

[bib63] Terwilliger T.C., Grosse-Kunstleve R.W., Afonine P.V., Moriarty N.W., Zwart P.H., Hung L.W., Read R.J., Adams P.D. (2008). Iterative model building, structure refinement and density modification with the PHENIX AutoBuild wizard. Acta Crystallogr. D Biol. Crystallogr..

[bib64] Uhlmann F. (2016). SMC complexes: from DNA to chromosomes. Nat. Rev. Mol. Cell Biol..

[bib65] Wells J.N., Gligoris T.G., Nasmyth K.A., Marsh J.A. (2017). Evolution of condensin and cohesin complexes driven by replacement of Kite by Hawk proteins. Curr. Biol..

[bib66] Wilhelm L., Bürmann F., Minnen A., Shin H.-C., Toseland C.P., Oh B.-H., Gruber S. (2015). SMC condensin entraps chromosomal DNA by an ATP hydrolysis dependent loading mechanism in Bacillus subtilis. eLife.

[bib67] Winn M.D., Ballard C.C., Cowtan K.D., Dodson E.J., Emsley P., Evans P.R., Keegan R.M., Krissinel E.B., Leslie A.G.W., McCoy A. (2011). Overview of the CCP4 suite and current developments. Acta Crystallogr. D Biol. Crystallogr..

[bib68] Wood A.J., Severson A.F., Meyer B.J. (2010). Condensin and cohesin complexity: the expanding repertoire of functions. Nat. Rev. Genet..

[bib69] Woods A., Sherwin T., Sasse R., MacRae T.H., Baines A.J., Gull K. (1989). Definition of individual components within the cytoskeleton of Trypanosoma brucei by a library of monoclonal antibodies. J. Cell Sci..

[bib70] Xu X., Nakazawa N., Yanagida M. (2015). Condensin HEAT subunits required for DNA repair, kinetochore/centromere function and ploidy maintenance in fission yeast. PLoS ONE.

[bib71] Yoshimura S.H., Hirano T. (2016). HEAT repeats - versatile arrays of amphiphilic helices working in crowded environments?. J. Cell Sci..

