# Supplementary material for: Structural Basis for a Safety-Belt Mechanism That Anchors Condensin to Chromosomes
Source: Cell. 2017 Oct 19;171(3):588–600.e24. doi: 10.1016/j.cell.2017.09.008 (PMC5651216; doi:10.1016/j.cell.2017.09.008)
Supplement: Document S1. Tables S1 and S2 [file mmc1.pdf]

Cell, Volume *171*

Supplemental Information

Structural Basis for a Safety-Belt Mechanism

That Anchors Condensin to Chromosomes

Marc Kschonsak, Fabian Merkel, Shveta Bisht, Jutta Metz, Vladimir Rybin, Markus Hassler, and Christian H. Haering

		S. pombe Cnd3–Cnd2 (SeMet-SAD)*	*S. pombe* Cnd3–Cnd2 (native)**	*S. cerevisiae* Ycg1–Brn1	*S. cerevisiae* Ycg1–Brn1 with DNA (I)	*S. cerevisiae* Ycg1–Brn1 with DNA (II)	*S. cerevisiae* Ycg1–Brn1$_{short\ kleisin\ loop}$ with DNA
**Data collection**	Space group	$P\ 2_1\ 2_1\ 2_1$	$P\ 2_1\ 2_1\ 2_1$	$P\ 3_1\ 2\ 1$	$P\ 2_1\ 2_1\ 2_1$	$P\ 2_1\ 2_1\ 2_1$	$P\ 2_1\ 2_1\ 2_1$
	Molecules/asu	2	2	2	1	1	1
	Cell dimensions (Å)						
	a	89.21	88.41	185.53	80.14	88.80	89.15
	b	141.76	142.11	185.53	114.83	117.94	116.22
	c	177.01	176.92	148.32	155.21	154.97	155.52
	Resolution (Å)	49.21-3.50 (3.71-3.50)	49.06-2.61 (2.67-2.61)	49.44-2.79 (2.94-2.79)	47.17-2.98 (3.14-2.98)	49.21-3.25 (3.43-3.25)	48.68-3.15 (3.32-3.15)
	R_{merge}	0.762 (5.147)	0.211 (1.482)	0.139 (1.745)	0.202 (1.416)	0.271 (1.854)	0.198 (1.115)
	$I/\sigma I$	13.6 (4.3)	11.2 (1.8)	12.9 (1.3)	8.5 (1.4)	6.7 (1.1)	8.4 (1.9)
	CC(1/2)	0.998 (0.907)	0.993 (0.678)	0.999 (0.444)	0.995 (0.565)	0.995 (0.513)	0.999 (0.287)
	Completeness (%)	99.9 (99.8)	100.0 (100.0)	100.0 (100.0)	100.0 (100.0)	100.0 (100.0)	100.0 (100.0)
	Redundancy	51.1 (48.0)	13.4 (13.0)	10.2 (10.4)	6.7 (7.1)	6.7 (6.8)	12.3 (8.6)
**Refinement**	Resolution (Å)		49.06-2.61	47.25-2.79	47.17-2.98	47.32-3.25	47.34-3.15
	No. reflections (total)		68,083	73,424	29,952	26,286	28,612
	R_{work}/R_{free}		0.22/0.26	0.20/0.25	0.22/0.26	0.24/0.27	0.25/0.28
	No. atoms						
	Protein		12,985	14,377	7,317	7,420	7,380
	Ligand/ion		0	0	753	768	732
	Water		47	32	0	0	0
	B-factors						
	Protein		75.31	80.71	62.87	85.51	67.28
	Ligand/ion		n.a.	n.a.	124.38	126.86	109.04
	Water		50.96	80.04	n.a.	n.a.	n.a.
	R.m.s deviations						
	Bond lengths (Å)		0.003	0.003	0.003	0.003	0.003
	Bond angles (°)		0.568	0.527	0.552	0.513	0.571

Table S1. Data collection and refinement statistics, related to Figures 2, 4 and 5.
Values in parentheses are for the highest-resolution shell, *from 4 merged datasets, **from 2 merged datasets.

				γ-kleisin	β-kleisin	HEAT
**Fungi**	Ascomycetes	Saccharomycetes	*Saccharomyces cerevisiae*	P38170	–	Q06680
			Kluyveromyces lactis	Q6CP87	–	Q6CQY6
		Sordariomycetes	*Chaetomium thermophilum*	G0SBJ6	–	G0SHK2
		Leotiomycetes	*Sclerotinia sclerotiorum*	A7F567	–	A7EQU0
		Eurotiomycetes	*Emericella nidulans*	Q5B5B5	–	Q5AUH4
		Dothideomycetes	*Phaeosphaeria nodorum*	Q0UXS3	–	Q0TYJ2
		Pezizomycetes	*Tuber melanosporum*	D5GHC6	–	D5G3W5
		Schizosaccharomycetes	*Schizosaccharomyces pombe*	Q9Y7R3	–	Q10429
	Basidiomycetes		*Moniliophthora roreri*	V2XXX7	–	V2YA02
	Microsporidians		*Encephalitozoon cuniculi*	Q8SWA2	–	M1JI11
**Animals**	Vertebrates	Mammals	*Homo sapiens*	Q15003	Q6IBW4	Q9BPX3
			Mus musculus	Q8C156	Q8BSP2	E9PWG6
		Birds	*Gallus gallus*	A0A1D5P3B2	XP004937454.2*	E1C181
		Reptiles	*Alligator mississippiensis*	A0A151MMA8	A0A151PFS9	A0A151P9P6
		Amphibians	*Xenopus laevis*	O13067	Q641G4	Q9YHB5
		Fish	*Danio rerio*	A1L231	Q5RH01	F1QJY8
		Cartilaginous fish	*Callorhinchus milii*	V9KIM3	V9KKJ5	V9KCC1
	Arthropods	Insects	*Apis mellifera*	A0A088AUC1	A0A088A774	A0A087ZT91
		Crustaceans	*Daphnia pulex*	E9GU42	E9GRW6	E9HM85
	Mollusks		*Octopus bimaculoides*	A0A0L8HZ58	A0A0L8FUD0	A0A0L8HTM5
**Plants**	Eudicots	Mustard family	*Arabidopsis thaliana*	Q564K3	Q9LUR0	Q0WVX3
		Mallow family	*Theobroma cacao*	A0A061FCQ2	A0A061EP02	A0A061EDQ5
		Pea family	*Glycine max*	I1KFG2	I1L0X2	I1MZF2
		Willow family	*Populus trichocarpa*	B9IAH8	B9HXR3	B9H3B9
		Grape family	*Vitis vinifera*	D7TEB5	F6H6W0	D7U7E6
		Nightshade family	*Solanum lycopersicum*	K4CF69	K4DHW3	K4D5U2
		Amaranth family	*Beta vulgaris*	A0A0J8FA76	A0A0J8BC64	A0A0J8CTW9
	Monocots	Grass family	*Zea mays*	B6U4T0	B6U1S5	K7W7V3
	Magnoliaphyta	Amborella family	*Amborella trichopoda*	W1NZC1	W1P0G4	W1NKW2
	Red algae		*Cyanidioschyzon merolae*	M1V4K2	M1VH11	M1VHT1
**Protists**	Choanoflagellates		*Monosiga brevicollis*	A9V7D8	A9V3Q2	A9UTE1
	Amoebozoa	Dictyostelium	*Dictyostelium discoideum*	Q54DR4	Q54DZ9	Q54TZ8
	Stramenopiles	Diatoms	*Phaeodactylum tricornutum*	B7G0X0	B7GDJ8	B7G8V9
		Oomycetes	*Saprolegnia parasitica*	A0A067CV32	A0A067CAZ5	A0A067BMH9
	Cryptomonads		*Guillardia theta*	L1INS6	L1I8P6	L1IG07

Table S2. Protein sequences used for sequence alignments, related to Figures 3B, 6A and S3.
Table lists Uniprot or *seq-ref identifiers.
